# Novel pan-cancer T cell exhaustion signature forecasts immunotherapy response and unveils BCAP31 in macrophages as a therapeutic target in neuroblastoma

**DOI:** 10.3389/fimmu.2025.1709225

**Published:** 2025-12-22

**Authors:** Shan Li, Jianjun Zhu, Xiang Huang, Fengming Ji, Jinrong Li, Zhigang Yao, Haoyu Tang, Ling Liu, Bing Yan, Chenghao Zhanghuang

**Affiliations:** 1 Department of Urology, Kunming Children’s Hospital (Children’s Hospital Affiliated to Kunming Medical University), Kunming, China; 2 Department of Urology, Children’s Hospital of Chongqing Medical University, Chongqing, China; 3 Department of Oncology, Kunming Children’s Hospital (Children’s Hospital Affiliated to Kunming Medical University), Kunming, China; 4 Department of Neonatology, Kunming Children’s Hospital (Children’s Hospital Affiliated to Kunming Medical University), Kunming, China; 5 Yunnan Key Laboratory of Children’s Major Disease Research, Yunnan Clinical Medical Center for Pediatric Diseases, Yunnan Province Clinical Research Center for Children’s Health and Disease, Kunming Children’s Solid Tumor Diagnosis and Treatment Center, Kunming Children’s Hospital (Children’s Hospital Affiliated to Kunming Medical University), Kunming, China

**Keywords:** machine learning, multi-omics analysis, neuroblastoma, pan-cancer analysis, T cell exhaustion

## Abstract

**Introduction:**

Gaining insights into the molecular features associated with T cell exhaustion (TEX) can offer fresh perspectives on predicting treatment responses, and we aim to investigate TEX-related tumor associated macrophages (TAM) subset to deeply understand underlying mechanisms of immune exhaustion.

**Methods:**

We performed pan-cancer single-cell RNA sequencing (scRNA-seq) and spatial transcriptomics RNA sequencing (stRNA-seq) analyses to investigate the subtype of TEX-associated TAMs, exploring its spatial distribution characteristics in context of immunotherapy. The pan-cancer scRNA-seq and RNA-seq datasets were incorporated to develop the STMN2+ Macrophage Signature (STMN2.SIG), which predicts immunotherapy response based on integrative machine learning techniques. Comprehensive scRNA-seq analysis, with *in vitro* experiments, investigated the mechanisms by which STMN2+ TAMs influence tumor progression and immune exhaustion.

**Results:**

A macrophage subset, STMN2+ TAMs, and an epithelial subtype, S phase Sympathoblasts were identified as TEX-related cellular subpopulations. A higher proportion of STMN2+ TAMs was observed in non-responders compared to responders in pan-cancer immunotherapy landscape. Pan-cancer STMN2.SIG performed well in predicting immunotherapy response in pan-cancer cohorts, potentially linked to intercellular interactions between STMN2+ TAMs and CD8+ Tex cells. stRNA-seq analysis confirmed that interactions and cellular distances between STMN2+ TAMs and CD8+ Tex cells impact therapy efficacy. In a co-culture system, silencing BCAP31 on TAMs drives CD8+ T cells toward an effector state in NB. And BCAP31 on TAMs is associated with modulation of JAK2-STAT3 pathway in tumor cells.

**Conclusion:**

Our study provides pan-cancer STMN2.SIG as an outperforming approach for patient selection of immunotherapy, and advances our understanding of TAM biology and suggests potential therapeutic strategies for downregulation of BCAP31 in TAMs.

## Introduction

Neuroblastoma (NB), the most prevalent extra-cranial cancer in children, originates in the sympathetic nervous system and is characterized by its heterogeneity (1). NB accounts for 7–8% of all pediatric cancers and is linked to roughly 15% of pediatric cancer-related fatalities (2). Advances in treatment have significantly bolstered survival rates among NB patients, with a current 5-year survival rate of 82.1% (3). NB cases are stratified into low-, intermediate-, and high-risk groups based on several factors: age at diagnosis, MYCN amplification status, International Neuroblastoma Staging System (INSS) stage, histological findings, and tumor cell ploidy (4). This categorization adheres to the 2000 COG risk criteria. While patients with low- and intermediate-risk NB enjoy near-100% survival rates, those with high-risk NB face a 5-year survival rate that hovers below 50%. High-risk NB patients also exhibit significant heterogeneity. Patients with a naturally favorable prognosis but classified as high-risk under the current system may receive overly aggressive treatments, exposing them unnecessarily to potential long-term adverse effects (5). Therefore, it is imperative to develop more precise markers to predict outcomes and guide therapy in NB.

Immunotherapy has demonstrated remarkable efficacy, leading to the acknowledgment of the tumor immune microenvironment (TIME) as a pivotal element in tumor progression, clinic prognosis, and treatment effectiveness (6). In recent years, the survival rate of high-risk NB cases has improved thanks to effective anti-GD2 therapy, highlighting the potential of immunotherapy in NB treatment (7). Despite NB’s low immunogenicity, which is attributed to its low mutational load and subsequent low neoepitope expression, as well as low MHC-I expression, the infiltration of T and NK cells into the tumor still serves as a prognostic indicator of survival (8). Beyond the inherent tactics that modified cells use to evade immune detection during NB development, another factor fueling NB proliferation is the compromised function of immune cells, which aligns with the creation of an immunosuppressive TIME (9). T-cell exhaustion (TEX), a form of immune cell dysfunction seen in various chronic infections and tumors (10), is characterized by a progressive loss of T cells’ capacity to carry out effector functions, such as cytokine production and cytotoxicity. Additionally, exhausted T cells exhibit an array of inhibitory receptors, disrupted metabolism, impaired memory recall, and diminished homeostatic proliferation (11). TEX is characterized by the hierarchical impairment of various pathways, including interferon-g (IFN-g), tumor necrosis factor (TNF), cytotoxic potential (CTL), and interleukin 2 (IL-2) production (12). The heterogeneity of TEX has been linked to the determination of distinct clinical outcomes and the effectiveness of immunotherapy in numerous adult malignancies (13). However, the heterogeneous TEX landscape in pediatric cancer, especially in NB, and its impact on clinical outcomes are still not well understood.

Several subsets of tumor associated macrophages (TAM) revealed by high-throughput sequencing have been found to exert diverse immune functions in TIME, interplaying with CD4+ T cells and CD8+ T cells to act as effector cells or exhaustion cells (14). We comprehensively utilized pan-cancer single-cell RNA sequencing (scRNA-seq) and spatial transcriptomics RNA sequencing (stRNA-seq) analyses to explore TEX-related macrophages and its influence on clinical prognosis and immunotherapy response. We then translated this finding into a clinically actionable signature. Given our focus on pediatric oncology and the critical need for better risk stratification of NB, which represents an aggressive pediatric malignancy with a complex TIME, so we sought to identify a signature associated with TEX to enhance the molecular clustering and prognosis stratification of NB patients.

## Materials and methods

### Integration of pan-cancer scRNA-seq cohorts

Twelve immunotherapy related datasets, containing both immunotherapy responsiveness records and scRNA-seq data, were incorporated. We obtained information of these cohorts from publicly accessible collections, covering skin cutaneous melanoma (SKCM, GSE120575 (15) and GSE115978 (16)), renal cell carcinoma (RCC, SCP1288 (17)), urothelial carcinoma (UC, GSE145281 (18)), triple negative breast cancer (TNBC, GSE169246 (19)), breast invasive carcinoma (BRCA, Bassez 2021 (20)), carcinoma of colon and rectum (CRC, GSE205506 (21) and GSE236581 (22)), non-small cell lung carcinoma (NSCLC, GSE207422 (23) and GSE243013 (24)), head and neck squamous cell carcinomas (HNSCC, GSE200996 (25)) and squamous cell carcinoma (SCC, GSE123813) and basal cell carcinoma (BCC, GSE123813 (26)). Cases showing partial response (PR) or complete response (CR) were defined as responders, and cases demonstrating progressive disease (PD) or stable disease (SD) were identified as non-responders.

### Acquisition of pan-cancer RNA-seq cohorts

We extensively gathered RNA-seq data and immunotherapy response characteristics from 16 immunotherapy datasets, covering 5 SKCM datasets (Hugo 2016 (27), Liu 2019 (28), Gide 2019 (29), Riaz 2017 (30), and Van Allen 2015 (31)), 4 RCC datasets (Braun 2020 (32), Ascierto 2016 (33), Powles 2017 (34) and Rini 2019 (35)), 2 UC datasets (Mariathasan 2018 (36) and Synder 2017 (37)), 1 glioblastoma multiform (GBM) dataset (Zhao 2019 (38)), 1 gastric cancer (GC) dataset (Kim 2018 (39)), and 3 NSCLC datasets (Jung 2019 (40), OAK 2017 (41), and POPLAR 2016 (42)). Transcriptomic data were log2-transformed, and batch influence across various cohorts were reduced through “sva” R package (43). We harnessed Braun 2020, Liu 2019, Gide 2019, Riaz 2017, OAK 2017 and Mariathasan 2018 as the training set to build ICS to forecast immunotherapy non-responders. Then Hugo 2016, Van Allen 2015, Synder 2017, Kim 2018, POPLAR 2016 and Zhao 2019 were designated as the internal validation set, while Ascierto 2016, Powles 2017, Rini 2019 and Jung 2019 were defined as the external validation set. We totally acquired six NB datasets: GSE137804 (scRNA-seq cohort), GSE49710 and GSE85047 sourced from GEO, TARGET-NB from TARGET, and E-MTAB-8248 and E-MTAB-179 from ArrayExpress. The molecular signature database (MSigDB) contains data on signaling pathways and marker genes related to TEX.

### Single cell transcriptome analysis

The scRNA-seq analysis was employed via “Seurat” R package (44), which offered cellular annotations and the separation of TAM sequencing data from every dataset. We sifted out low-quality cells of >40,000 UMI per cell, <500 genes per cell, >5,000 genes per cell and >20% mitochondrial genes. We excluded doublets through “DoubletFinder” R package (45). We integrated pan-cancer scRNA-seq cohorts and reduced batch influence through “Harmony” R package (46). Utilizing canonical maker genes of TAM subpopulations (47), various subtypes of TAMs were defined. Subpopulations within the TAM cluster were named according to their significantly and specifically expressed marker gene. Differential expression genes between responders and non-responders were calculated through “FindMarkers” function, with criteria of log2 fold change (log2FC) > 0 and adjusted p-value < 0.05. We employed “Monocle” R package to develop cellular development trajectories, demonstrating the placement of responders and non-responders through the trajectory lineages (48). Seven scRNA-seq rating algorithms (AUCell in “AUCell” R package, Ucell in “Ucell” R package, ssGSEA and GSVA in “GSVA” R package, singscore in “singscore” R package, AddModuleScore and PercentageFeatureSet in “Seurat” R package) were harnessed to calculate enrichment scores (44, 49–51). Functional enrichment analysis within Gene Ontology (GO), Kyoto Encyclopedia of Genes and Genomes (KEGG) and WikiPathway terms in scRNA-seq datasets was employed through “SCP” R package (https://github.com/zhanghao-njmu/SCP). “CellChat” R package was harnessed to compute cellular interplay networks across cellular subpopulations (52). “pySCENIC” method was harnessed to compute enriched transcription factors (TF) and regulon activities of cellular phenotypes, constructing TF regulatory networks and determining steady cellular conditions (53).

### Spatial transcriptome analysis

To elucidate biological roles of TAMs in immunotherapy within stRNA-seq resolution, we accessed stRNA-seq data of hepatocellular carcinoma (HCC) cases receiving PD-1 blockade treatment (54). To reliably determine the cellular compositions at each site on 10x stRNA-seq slides, deconvolution analyses was applied to obtain the cellular compositions (55), which was based on stRNA-seq and scRNA-seq data of the same cancer. Concurrently, to identify the spatial locations, we conducted an integrated analyses of scRNA-seq and stRNA-seq data through “CellTrek” R package (56). The spatial k-distance across various TAM phenotypes and cellular subpopulations was analyzed through run_kdist function. We assessed the spatial k-distance among different cellular subpopulations within tissue sections, arranged them from nearest to farthest, and then applied the robust rank aggregation (RRA) method to derive a final ranking of cell types (57). To explore the dynamic biological processes and spatial trajectories across high-density regions of various cell subpopulations at a spatial resolution, “SPATA2” R package was employed (58). Additionally, we harnessed “SpaTalk” R package to detect intercellular communication modes among the spatial coordinates (59).

### Model construction and calculation of risk score

Leveraging marker genes of TEX-related TAMs, we developed the pan-cancer STMN2.SIG to forecast responsiveness to immunotherapy across pan-cancer RNA-seq datasets, as well as NB-specific STMN2.SIG to predict prognosis in NB patients. While the gene set for model construction is constant, the algorithm and output for immunotherapy prediction and neuroblastoma prognosis are tailored to their respective clinical questions. We harnessed twelve ML approaches covering random forest (RF), Lasso, Ridge, elastic net (Enet), stepwise Glm, GlmBoost, linear discriminant analysis (LDA), partial least squares regression for Glm (plsRglm), generalized boosted regression modeling (GBM), extreme gradient boosting (XGB), support vector machine (SVM) and Naives Bayes to forecast immunotherapy effectiveness. To prevent overfitting, we employed nested resampling, which involved a two-tiered k-fold cross-validation process: one for optimizing model hyperparameters and another nested within it for model selection. Meanwhile, we utilized a 1000-evaluation random search across a 5-fold cross-validation framework, repeated five times for each model. We then selected the top-performing models from three different strategies and combined them to create an ensemble model. We integrated 10 prognosis ML algorithms, covering random survival forest (RSF), elastic network (Enet), Lasso, Ridge, stepwise Cox, CoxBoost, partial least squares regression for Cox (plsRcox), supervised principal components (SuperPC), generalized boosted regression modeling (GBM), and survival support vector machine (survival-SVM) for NB prognosis prediction. A sum of 113 predictive ML combinations and 101 prognosis ML combinations was assessed in the training set to construct model with help of leave-one-out cross-validation (LOOCV) system. Signatures with fewer than five model genes were excluded. Average area under the curve (AUC) and C-index of ML combinations was obtained across the train, internal test and external test datasets (60), with the combination scoring best average AUC or C-index defined as the best model. The risk scores were obtained by direct output of the final trained ML models. To extensively assess the predictive capabilities of pan-cancer STMN2.SIG, thirteen immunotherapy associated signatures were encompassed (Stem.Sig (61), IMS.Sig (62), CRMA.Sig (63), ImmmunCells.Sig (64), IFNG.Sig (65), T.cell.infamed.Sig (65), PDL1.Sig (66), TcellExc.Sig (16), NLRP3.Sig (67), Cytotoxic.Sig (68), TRS.Sig (69), LRRC15.ICS (70), IMPRES.Sig (71), and IPRES.Sig (27)) to benchmark their results. The codes and algorithms linked to these signatures were sourced from the original study. In scRNA-seq data, we pinpointed upregulated and downregulated genes in non-responders and calculated the risk score using the specified formula: Risk score = Algorithm_Score (up-regulated) – Algorithm_Score (down-regulated).

### Model verification in precision, stability and reliability

To rigorously evaluate the precision, robustness, and reproducibility of the model, a detailed array of validation approaches was implemented. The model’s precision was tested through confusion matrix by “cvms” R package. Moreover, Receiver Operating Characteristic (ROC) curves, calibration curves, and decision curve analysis (DCA) were harnessed to measure the model’s accuracy, discriminative capability, and clinic benefits. The model’s prediction efficacy was assessed in comparison to traditional clinical features by ROC analysis. Additionally, univariate and multivariate logistic regression analyses were performed to validate the model’s independently predictive capability. Ultimately, a nomogram was developed to visually illustrate the predictive strength of the model.

### Function enrichment analysis

Differentially expressed genes (DEGs) were recognized by comparing two subgroups separated by the median expression of a certain gene. DEGs were computed through “limma” R package, based on False-discovery rate (FDR) <0.05 and absolute log2fold change (FC) >1. The functional enrichment of DEGs was employed within GO and KEGG via “clusterprofiler” R package (72). The ssGSEA technique on each patient was implemented through “GSVA” R package (51), within the “h.all.v7.4.symbols.gmt” gene set from MSigDB. We harnessed GSEA to investigate the signaling pathways related to differential subgroups (73), within the criteria of p < 0.05 and Normalized Enrichment Score (NES) > 1.

### Isolation of primary cells and co-culture experiments

Human primary tumor cells were collected from HLA-A2+ patient and obtained by surgical procedures approved by the Ethics Committee of the Children’s Hospital affiliated to Kunming Medical University, with informed consent acquired from patients’ guardians. The tissues were washed three times with PBS buffer, cut into small pieces (~ 1mm³), and digested with 1 mg/mL type IV collagenase at 37°C for 2 hours. After digestion, a single-cell suspension was retrieved by a 200-mesh filter and was then cultured in DMEM medium supplemented with 10% fetal bovine serum to acquire adherent cells. Peripheral blood mononuclear cells (PBMCs) were obtained from HLA-A2+ patient blood samples through the MACSpread PBMC Isolation Kit (130-115-169, Miltenyi). Human primary TAMs were acquired from CD14+ cells (CD14 Microbeads, Miltenyi Biotec) of HLA-A2+ patients’ blood after 6 days of culture in presence of M-CSF (50 ng/mL Peprotech) (74). Human primary CD8+ T cells were isolated and purified through the Dynabeads Untouched Human CD8 T Cell Assay Kit (11348D, Thermo Fisher Scientific Inc.), based on the manufacturer’s instructions. Samples with a purity more than 95%, as validated through flow cytometry, were utilized in following experiments. Primary cancer cells and TAMs were seeded into 12-well plates, and activated CD8+ T cells were then added to the co-cultures. Flow cytometry was utilized to evaluate the cytotoxic functions of CD8+ T cells.

### Function experiments of tumor cells

NB cell lines SK-N-SH, and SK-N-BE(2) were obtained from Cell Collection of the Chinese Academy of Sciences (China) and cultured with high-glucose DMEM (MeilunBio, MA0212, Dalian, China) containing 10% fetal bovine serum (Corning, 35-076-CV, Corning, NY, USA), incubating at 37 °C in an incubator containing 5% CO2. The siRNAs for BCAP31 and corresponding negative controls were produced by Tsingke Biotechnology (China). The sequences of si-BCAP31 were illustrated in **Supplementary Table S1**. RT-qPCR were utilized to verify the silence effects for BCAP31, with primers supplied in **Supplementary Table S2**. CCK8 assays (MCE, HY-K0301), EdU assays (Beyotime Biotech, Beijing, China), scratch wound healing assays, and Transwell assays (Falcon, 353097, USA and Biozellen, B-P-00002-4, China) were employed to explore the cell viability, migration, and invasion with the manufacturers’ instructions. Cell cycle experiments and apoptosis assays were implemented by flow cytometry with the BD detection kit.

### Flow cytometry

In co-culture experiments, cells were resuspended in ice-cold PBS for flow cytometry staining. For intracellular staining of IFN-γ (BioLegend, 505830) and Granzyme B (GrzB; BioLegend, 372208) in T cells, the cells were stimulated at 37°C with 5% CO_2_ for 4 hours in the presence of ionomycin calcium salt (100 ng/mL), monensin sodium salt (1.5 mg/mL), and phorbol 12-myristate 13-acetate (PMA; 100 ng/mL), followed by flow cytometry staining processing. Prior to surface or intracellular staining, cells were incubated with Zombie Aqua™ Fixable Viability Dye (1:400, BioLegend) on ice for 30 minutes to exclude dead cells. For surface staining of isolated tumor-infiltrating lymphocytes or *in vitro* cultured cells, samples were incubated in FACS buffer (1× PBS containing 2% FBS) with Fc receptor blocking solution (Purified anti-human IgG Fc Antibody, 1:100, BioLegend, 410701) to minimize nonspecific binding. After Fc blockade, cells were incubated with appropriate fluorophore-conjugated antibodies on ice for 30 minutes, covering CD45-APC-Cy7, CD3-BV510, CD8-AF488, IFN-γ-PE-Cy7, TNF-α-PerCP-Cy5.5, and Perforin-PE (details of the antibody panel listed in **Supplementary Table S3**). After staining, samples were fixed with 2.1% formaldehyde on ice for 15 minutes, filtered through a 70 μm cell strainer, and analyzed by the BD FACSCanto II flow cytometer and Beckman CytoFLEX. Data were processed through FlowJo software.

## Results

### Pan−cancer scRNA-seq atlas of immunotherapy

**Figure 1:**
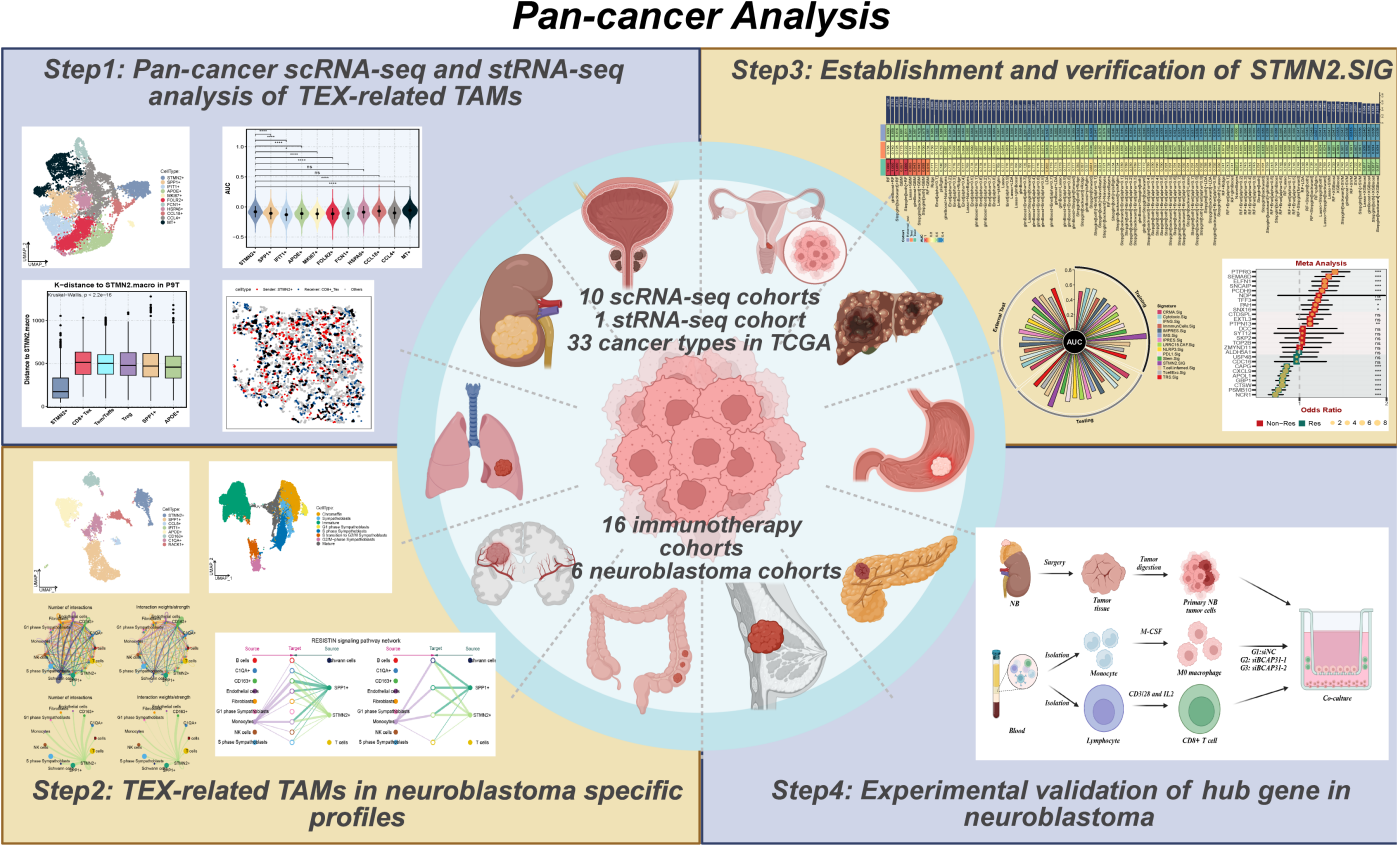
Workflow summary. This figure illustrates the workflow of the study, exploring the roles of TEX in pan-cancer immunotherapy landscape and NB specific context, based on integrated scRNA-seq and stRNA-seq analyses. The study used machine learning to construct the model for predicting immunotherapy response and NB patients’ prognosis. BCAP31 was identified as a hub gene for TEX-related TAMs, and its role was demonstrated through experiment validations. TEX, T cell exhaustion; NB, neuroblastoma; TAM, tumor associated macrophages.

**Figure 2:**
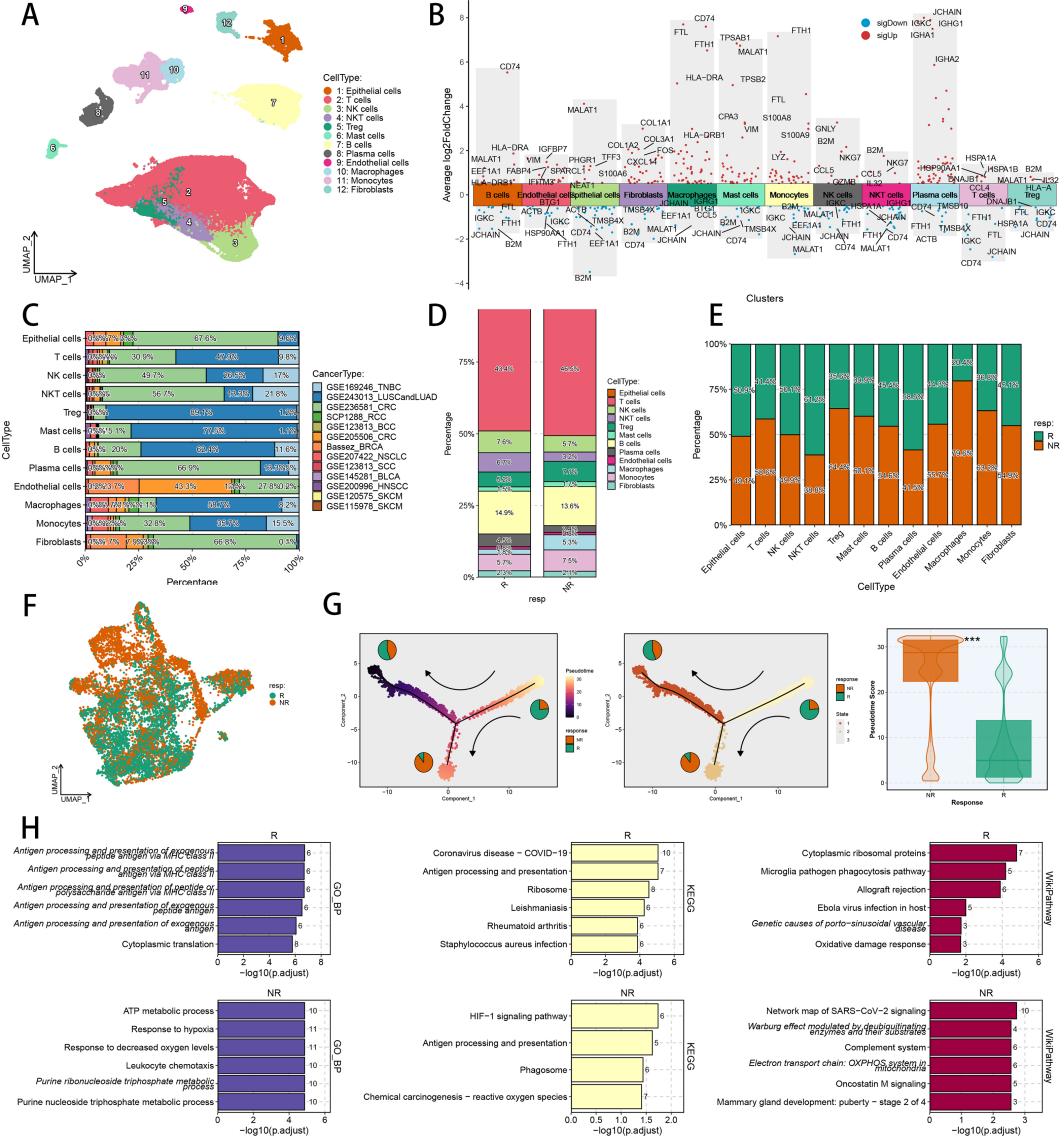
Major cell type landscapes of immunotherapy-related pan-cancer scRNA-seq cohorts. **(A)** Color-coded UMAP plot of major cell types in immunotherapy-related scRNA-seq cohorts. **(B)** Marker genes of each major cell type. **(C)** The cell proportion of each major cell type in every scRNA-seq cohort. **(D)** The proportion of each major cell type between responders and non-responders. **(E)** The proportion of responder cells and non-responder cells in each major cell type. **(F)** The cell distribution of immunotherapy responders and non-responders in TAMs. **(G)** Trajectory analysis of TAMs from immunotherapy responders and non-responders. **(H)** Biological Process of GO enrichment, KEGG analysis and WikiPathway enrichment analyses of DEGs between TAMs from immunotherapy responders and non-responders. UMAP, uniform manifold approximation and projection; KEGG, Kyoto Encyclopedia of Genes and Genomes; DEGs, differential expressed genes; GO, Gene Ontology.

The work diagram of our pan-cancer research is illustrated in **Figure 1**. Ten scRNA-seq cohorts with immunotherapy responsiveness records were harnessed to investigate the relationship between TAMs and immunotherapy reaction. To characterize various major cell types, we utilized uniform manifold approximation and projection (UMAP) to employ dimensionality reduction and clustering of single cells covering ten cancer types (**Figure 2A**). Twelve major cell types were distinguished in the integrated dataset, and the typical marker genes of cell types confirmed the successful annotation of these populations (**Figure 2B**). Concerning immunotherapy responsiveness, the cell projections and total cell counts in responders and non-responders, as determined by scRNA-seq, varied significantly across each phenotype (**Supplementary Figure S1A**) and each cancer cohort (**Figure 2C**). We analyzed immune evasion and immune checkpoint genes across major cell types, demonstrating the considerable expression of CCL2, TGFB1, IL10RB, and TGFBR1 in fibroblasts (**Supplementary Figure S1B**). Across the various cell types, TNBC, BRCA, NSCLC, and CRC made up the largest shares, and TAMs were found in every cancer dataset (**Figure 2C**), indicating their pervasive presence in tumor tissues undergoing ICI treatment. To explore the complexities of TIME in responders and non-responders, we assessed the ratio of major cell types in responders and non-responders (**Figures 2D, E**). We found that the proportion of TAMs was markedly lower in responders, suggesting potential mechanisms and functions that might influence immunotherapy responsiveness in cancer patients. To further investigate the impact of TAMs on immunotherapy efficacy, we subsequently applied UMAP to reduce the dimensions of TAMs. This analysis revealed that TAMs from responders and non-responders were clearly separated, indicating their potential influence on immunotherapy responsiveness (**Figure 2F**). Additionally, pseudotime analysis indicated that TAMs can branch into various subpopulations throughout tumor progression. TAMs from responders and non-responders were notably divided into two distinct groups, each with significantly varied pseudotime scores, suggesting that these cells follow divergent differentiation trajectories in responders and non-responders (**Figure 2G**; **Supplementary Figure S1C**). To delve into the biological disparities between responders and non-responders, we assessed the gene expression profiles in TAMs and found several DEGs, such as IFI6, SPP1 and BAG3, with markedly different expression profiles (**Supplementary Figures S1D, E**). Furthermore, we employed functional enrichment analyses with Biological Process of GO, KEGG and WikiPathway, focusing on DEGs. The upregulated genes in responders were significant in “Antigen processing”, “Ribosome” and “Oxidative damage response”, while the upregulated genes in non-responders were significant in “ATP metabolic process”, “HIF-1 signaling pathway” and “Warburg effect” (**Figure 2H**).

### TAM heterogeneity in pan-cancer scRNA-seq cohorts

**Figure 3:**
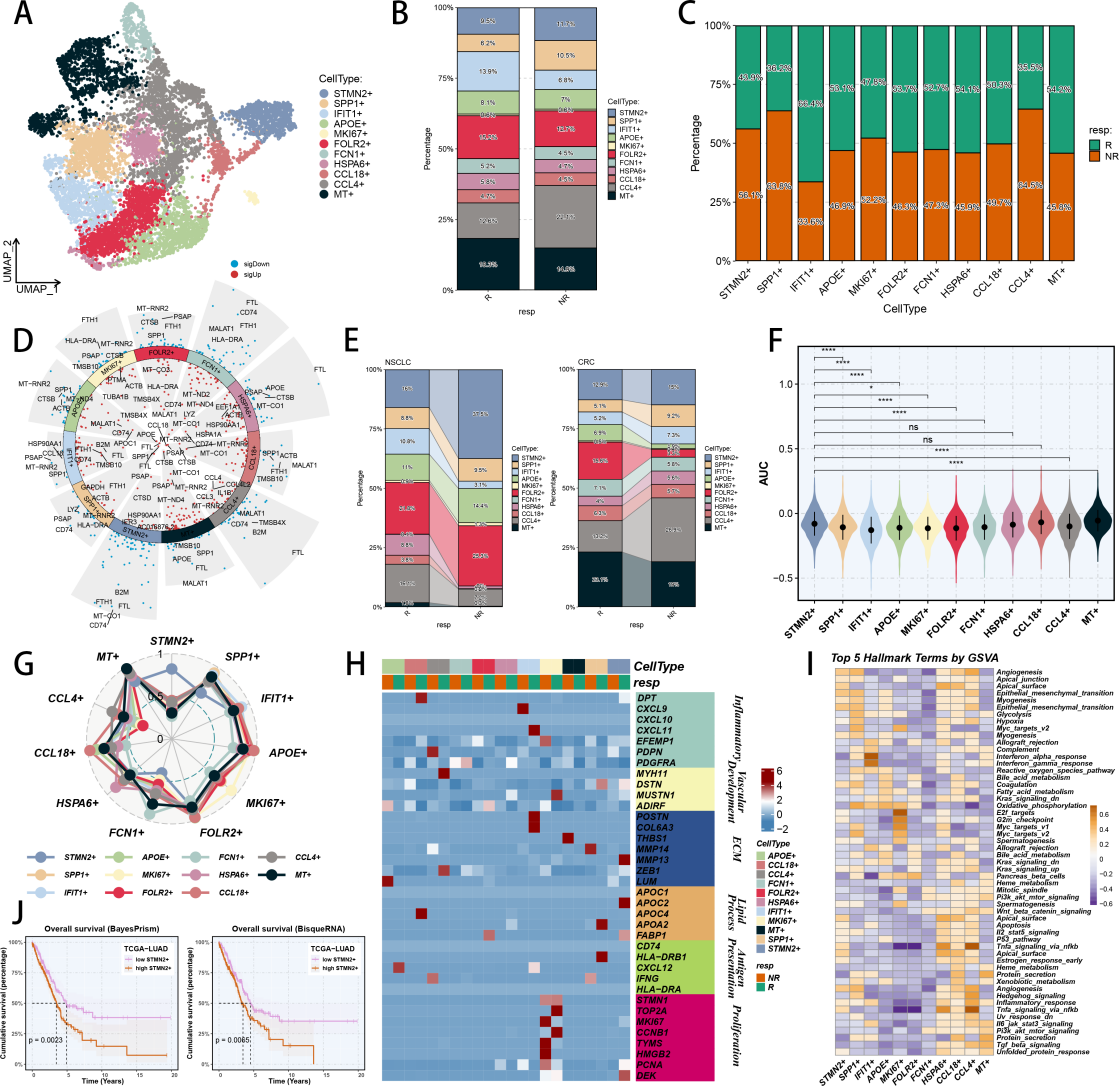
Pan-cancer TAM heterogeneity and identification of TEX-related TAMs in immunotherapy. **(A)** Colour-coded UMAP plot of TAM subgroups. **(B)** The proportion of TAM subgroups in immunotherapy responders and non-responders. **(C)** The proportions of immunotherapy responders and non-responders in each TAM subgroup. **(D)** Marker genes of each TAM subtype. **(E)** The proportion of each TAM subgroup in immunotherapy responders and non-responders of CRC and NSCLC. **(F)** Enrichment scores calculated by ssGSEA algorithm of each TAM subset based on TEX-related genes. **(G)** Radar plot showing the mean expression of TAM subgroup signatures (top 15 marker genes) calculated by the AUCell algorithm in the reclassified TAM subtypes. **(H)** Heatmap showing the mean expression of associated gene signatures in each TAM subtype. **(I)** Hallmark terms of differentially expressed genes significantly enriched in each TAM subtype by the AUCell algorithm. The colors represent the scaled value of the −log10 p value. **(J)** Survival analysis of high STMN2+ TAM group and low STMN2+ TAM group divided by BisqueRNA and BayesPrism deconvolution analysis. *p < 0.05; **p < 0.01; ***p < 0.001; ****p < 0.0001.

Afterwards, we researched the heterogeneity of TAMs within pan-cancer immunotherapy condition and ultimately defined eleven TAM sub types (**Figure 3A**). On the basis of literature review, we successfully defined differential TAM phenotypes which notably expressed hub genes of SPP1, APOE, FOLR2 and STMN2 (**Supplementary Figure S2A**), and depicted their functional annotations (**Supplementary Figure S2B**) (75, 76). Upon accurate phenotype identification, it was observed that the prevalence of STMN2+ TAM, SPP1+ TAM and CCL4+ TAM was significantly elevated in non-responders, indicating a potentially crucial role in the response to immunotherapy (**Figure 3B**). Additionally, non-responders exhibited higher numbers and proportions of STMN2+ TAM, SPP1+ TAM, MKI67+ TAM and CCL4+ TAM (**Figure 3C**). Moreover, typical marker genes of each TAM subtype confirmed the successful annotation of these populations (**Figure 3D**). Next, we examined the differences in TAM subpopulation proportions between responders and non-responders in CRC and NSCLC cohorts, indicating a higher proportion of STMN2+ TAM and SPP1+ TAM among non-responders (**Figure 3E**). Hence, we utilized scRNA-seq scoring method within every TAM phenotype through AUCell technique based on TEX markers. The results showed that STMN2+ TAM had the highest scores, suggesting that they may have a close association with TEX (**Figure 3F**). We also calculated the mean expression of TAM subgroup signatures (top 15 marker genes) by AUCell algorithm to consolidate our annotation (**Figure 3G**). And the mean expression of associated genes in each TAM subtype was illustrated to depict their probably biological functions (**Figure 3H**). Hallmark signaling signature was utilized to perform enrichment analysis by AUCell algorithm within each TAM phenotype, indicating the probably oncogenic roles of TAM sub types (**Figure 3I**). Additionally, we examined the expression profiles of immune checkpoint genes within every TAM phenotype, and found that CSF1R and IL10RB expressed significantly higher in STMN2+ TAM (**Supplementary Figure S2C**). In addition, another four scRNA-seq scoring algorithms further confirmed the highest TEX scores for STMN2+ TAM (**Supplementary Figure S2D**). Deconvolution analysis based on BisqueRNA and BayesPrism algorithms was also harnessed to integrate scRNA-seq and RNA-seq data to reveal STMN2+ TAM’s indication of poor prognosis. Deconvolution techniques could compute the cellular abundance of STMN2+ TAM in RNA-seq data with follow-up information based on scRNA-seq annotations, which demonstrated that LUAD patients with high STMN2+ TAM abundance suffered a poorer OS (**Figure 3J**). Meanwhile, we investigated the transcription factor (TF) modes of each TAM phenotype and concluded that expressions of IRF3 and TEAD4 regulons were abundant in STMN2+ TAM (**Supplementary Figure S2E**).

### Interplay of TAMs and CD8+ Tex cells affects immunotherapy response

**Figure 4:**
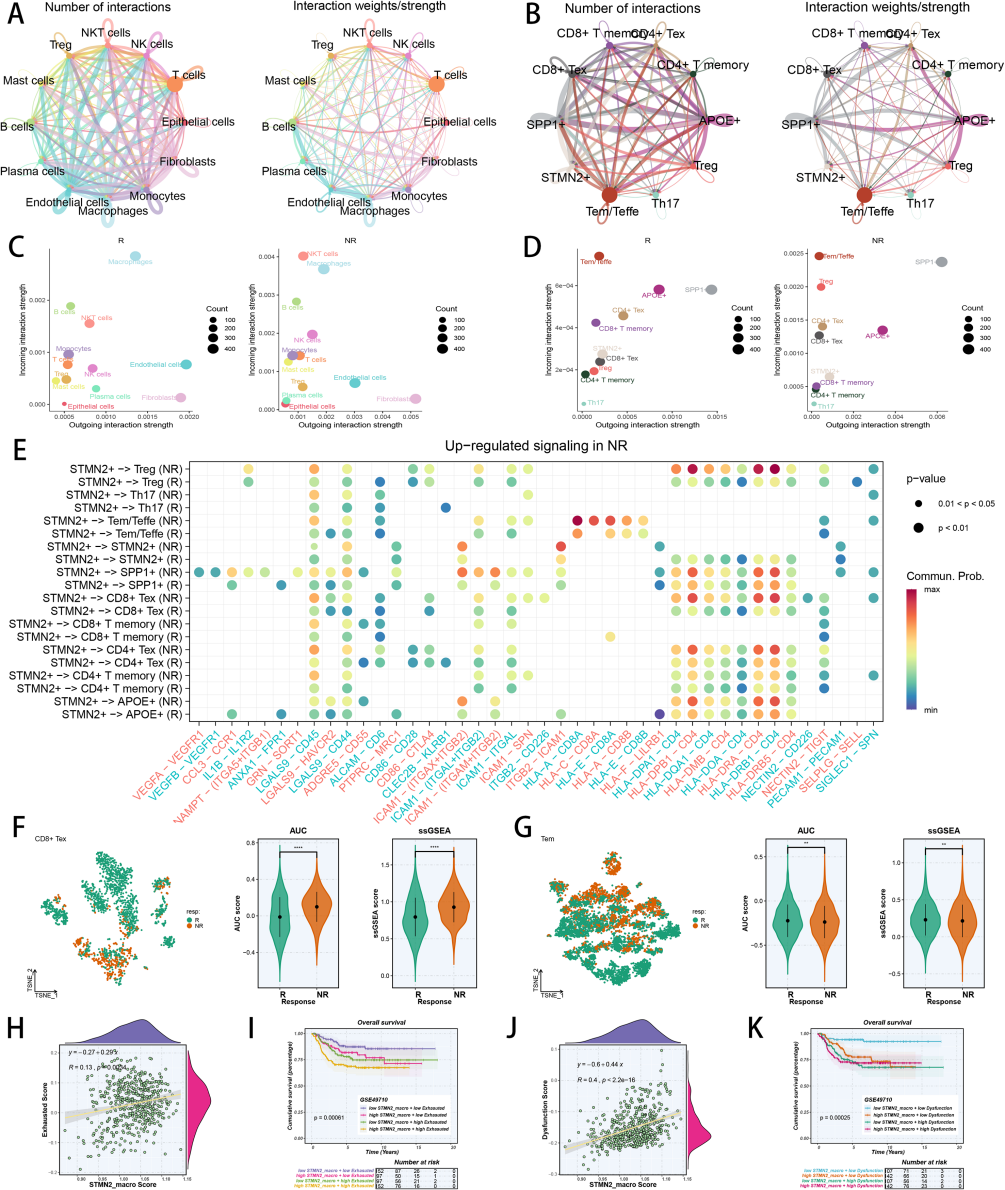
The communication between STMN2+ TAM and CD8+ Tex cells significantly influence immunotherapy response. **(A)** Cell-cell interactions among major cell types. **(B)** Cell–cell interactions among TAM subpopulations and different T cell subpopulations. **(C)** The relationship between differential outgoing interactions and incoming interaction strength for different major cell types. **(D)** The relationship between differential outgoing interactions and incoming interaction strength for different TAM subpopulations and different T cell subpopulations. **(E)** Ligands and receptors for signal communication among STMN2+ TAM and different T cell subpopulations. **(F)** Color-coded TSNE plot of responders and non-responders in CD8+ Tex cells, and comparison of exhausted scores of CD8+ Tex cells between immunotherapy responders and non-responders by AUCell and ssGSEA algorithm. **(G)** Color-coded TSNE plot of responders and non-responders in Tem cells, and comparison of exhausted scores of Tem cells between immunotherapy responders and non-responders by AUCell and ssGSEA algorithm. **(H)** Spearman correlation of Exhausted score with STMN2+ TAM risk score in GSE49710 cohort. **(I)** Survival analysis of distinct Exhausted score and different STMN2+ TAM risk score in GSE49710 cohort. **(J)** Spearman correlation of Dysfunction score with STMN2+ TAM risk score in GSE49710 cohort. **(K)** Survival analysis of distinct Dysfunction score and different STMN2+ TAM risk score in GSE49710 cohort. *p < 0.05; **p < 0.01; ***p < 0.001; ****p < 0.0001.

To elucidate how TAMs influence the immunotherapy efficacy across various cancers, we examined the interactions among key cell types by analyzing unique cellular communication pathways. Our analyses indicated that TAMs and T cells were the most actively involved in intercellular communication (**Figure 4A**). We then explored the intercellular communication between these T cell subpopulations and TAMs, demonstrating that SPP1+ TAM and T effector memory/T effector (Tem/Teff) cells were highly engaged in intercellular interactions (**Figure 4B**; **Supplementary Figure S3A**). Specifically, we found that TAMs had higher incoming communication signals in responders and non-responders (**Figure 4C**), whereas SPP1+ TAM and Tem/Teff cells were mostly engaged in responders and non-responders (**Figure 4D**). Additionally, we found that interactions of HLA-DRB1-CD4 between STMN2+ TAM and CD8+ Tex cells, as well as HLA-A-CD8A between STMN2+ TAM and Tem/Teff cells, were particularly strong and could influence immunotherapy effectiveness (**Figure 4E**; **Supplementary Figure S3B**). In CD8+ Tex cells, we identified several DEGs between responders and non-responders, such as HLA-DRB1, MALAT1, and CYBA (**Supplementary Figures S3C, D**). To examine the function of CD8+ Tex cells in immunotherapy, we used T-distributed stochastic neighbor embedding (t-SNE) to visualize their distribution patterns in responders and non-responders (**Figure 4F**), and obtained the exhausted score via AUCell and ssGSEA algorithm (**Figure 4F**; **Supplementary Figure S3E**). We subsequently noted that CD8+ Tex cells from responders exhibited lower exhaustion scores compared to those from non-responders (**Figure 4F**). In parallel, we assessed the effector scores of Tem/Teffe cells and found that responders had more effector scores than non-responders (**Figure 4G**; **Supplementary Figure S4F**). Overall, we hypothesized that CD8+ Tex cells might contribute to non-responsiveness to immunotherapy, while Tem/Teffe cells could enhance immunotherapy responsiveness. This suggests that the interplay between STMN2+ TAM and CD8+ Tex cells may play a role in determining immunotherapy response. Following this, we delved deeper into the relationships between STMN2+ TAM and CD8+ Tex cells specifically in the context of NB and calculated the exhaustion score and STMN2+ TAM score through ssGSEA. Notably, we found that both exhaustion scores and dysfunction scores were significantly positively correlated with STMN2+ TAM scores (**Figures 4H, J**). We then stratified NB patients into four subgroups according to the median values of exhaustion score, dysfunction score, and STMN2+ TAM score. Our analysis revealed that patients with high exhaustion scores and high STMN2+ TAM scores had the worst prognosis (P < 0.05), as did patients with high dysfunction scores and high STMN2+ TAM scores (P < 0.05, **Figures 4I, K**). These findings suggested that STMN2+ TAM may interact with CD8+ Tex cells to influence both immunotherapy responsiveness and the prognosis of NB patients.

### Spatially single-cell location characteristics

**Figure 5:**
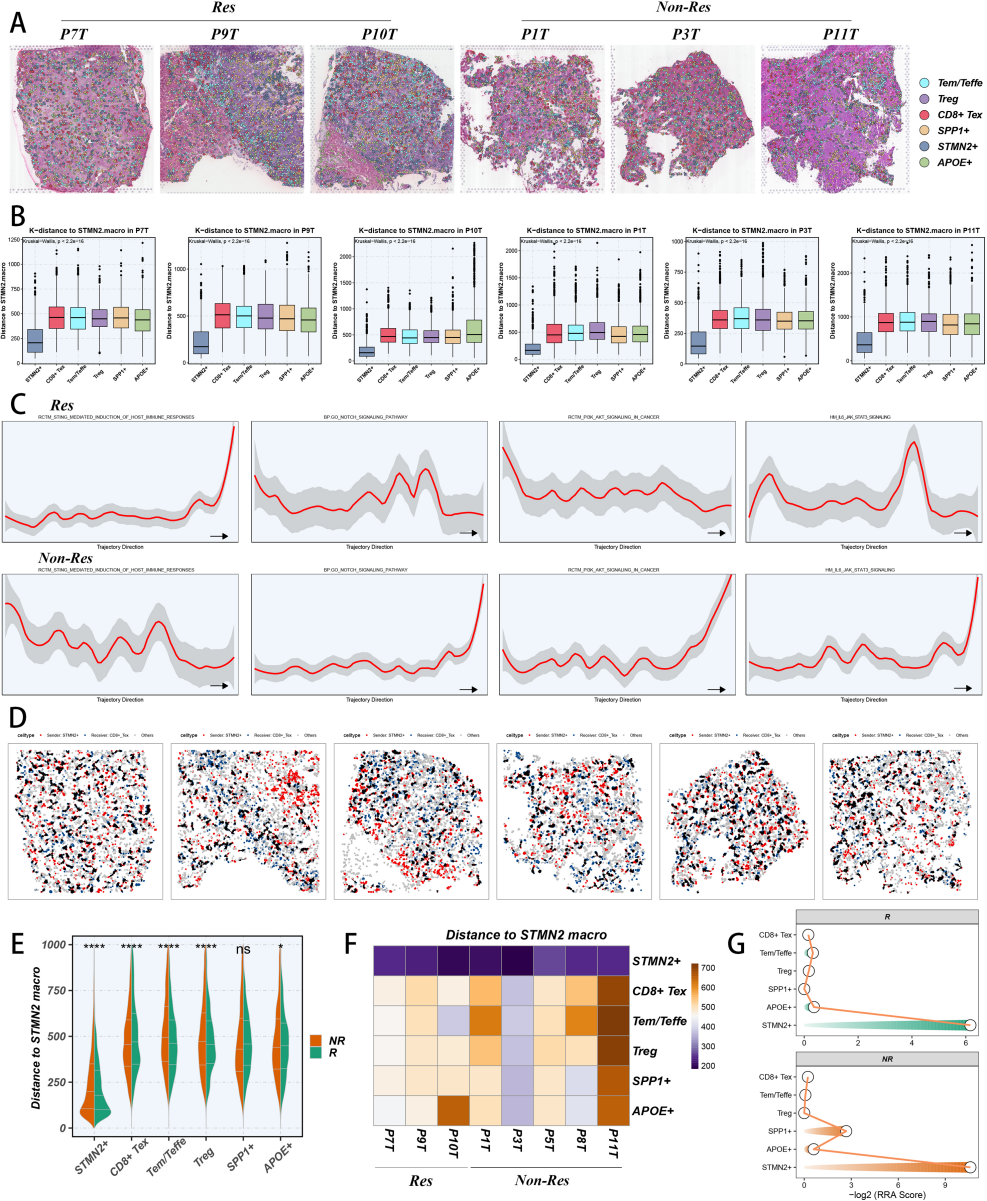
Spatial distribution characteristics of TAM subpopulations and T cell subpopulations. From left to right: P7T, P9T and P10T were responders, P1T, P3T and P11T were non-responders. **(A)** Spatial cell charting and location of TAM subpopulations and T cell subpopulations via CellTrek analysis in tumor sections. **(B)** Spatial k-distance to STMN2+ TAM among TAM subpopulations and T cell subpopulations across tumor sections. **(C)** Changes in the specific pathway activity of cell subtypes along the trajectory direction of Tem/Teffe shifting to CD8+ Tex. **(D)** Spatial distribution of the LGALS1-PTPRC interaction in tumor sections. **(E)** Violin plot showing the spatial k-distance to STMN2+ TAM in responders and non-responders. **(F)** Heatmap showing the average k-distance from different cell types to STMN2+ TAM in each tumor slice. The columns were scaled. **(G)** Integrated ranking of cell types based on proximity to STMN2+ TAM using RRA algorithm in responders and non-responders. The smaller the RRA score of a certain cell type, the closer it is to STMN2+ TAM. *p < 0.05; **p < 0.01; ***p < 0.001; ****p < 0.0001.

To inspect deeper into the spatial location patterns of TEX-related subpopulations in immunotherapy responders and non-responders, we obtained scRNA-seq and stRNA-seq data from eight HCC patients undergoing ICI therapy (five non-responders and three responders) (54). Following quality control (**Supplementary Figure S4A**), we used unbiased clustering and spot features to annotate the tumor section spots in accordance with the annotations provided in the literature (**Supplementary Figure S4B**). We subsequently employed CellTrek to directly locate individual cells to their corresponding spatial locations in tumor sections using integrated scRNA-seq and stRNA-seq data from the same patient (56). Unlike conventional stRNA-seq deconvolution techniques, the framework translates stRNA-seq coordinates to the single-cell level, thus reconstructing high-resolution spatial single-cell atlases (**Figure 5A**). The CellTrek mapping analyses effectively visualized the spatial distribution of SPP1+ TAM, STMN2+ TAM, APOE+ TAM, Tregs, CD8+ Tex cells, and Tem/Teffe cells within tumor sections, revealing their substantial infiltration in TIME (**Figure 5A**). Subsequently, we examined the spatial k-distance between TAM subpopulations and T cell subtypes across each tumor section, showing that in responders, STMN2+ TAM were in closer proximity to Tem/Teffe cells, whereas in non-responders, STMN2+ TAM were closer to CD8+ Tex cells (**Figure 5B**). To investigate the spatial dynamics from high-density Tem/Teffe regions to CD8+ Tex regions, we mapped the high-density spots of various cell types and conducted spatial trajectory analysis in tumor sections. We initially applied the “UCell” algorithm to conduct enrichment analysis in stRNA-seq data using the marker genes of each cell subpopulation, thereby identifying the high-density regions of each cell subpopulation (**Supplementary Figure S4C**). Next, we delineated the potential spatial trajectories of Tem/Teffe cells transitioning to CD8+ Tex cells based on the identified high-density cell regions. Utilizing these trajectories, we conducted spatial trajectory analysis, which uncovered variable presences of some signaling pathways (**Figure 5C**). We found STING signaling pathway with an activated state in responders, while it demonstrated an unsteady condition in non-responders (**Figure 5C**), suggesting the probable activation of STING signaling pathway for the sake of immunotherapy response. Additionally, the activities of the IL6_JAK_STAT3, NOTCH, and PI3K-AKT signaling pathways were unstable in responders, while they exhibited a slight upward trend in non-responders, suggesting their potential activation in non-responders (**Figure 5C**). Furthermore, we performed Spatalk analysis to elucidate the spatial communication patterns between STMN2+ TAM, CD8+ Tex cells and Tem/Teffe cells in tumor sections, investigating the ligand-receptor interactions (LRIs) between LGALS1 and PTPRC (**Figure 5D**). Our results raised that the interaction distances of the LGALS1-PTPRC LRI between STMN2+ TAM and CD8+ Tex cells were closer in non-responders (**Supplementary Figure S5A**). And the interaction distances of the LGALS1-PTPRC LRI between STMN2+ TAM and Tem/Teffe cells were closer in responders (**Supplementary Figure S5B**). We also examined other LRIs associated with immunosuppression in tumor sections from both responders and non-responders. Using the RRA method, we generated a comprehensive ranking of these LRIs for each group (**Supplementary Figure S5C**). Subsequently, by integrating the cell-to-cell k-distance across all tumor sections, we assessed the spatial k-distance to STMN2+ TAM in both responders and non-responders. Our analysis indicated that in responders, STMN2+ TAM were in closer proximity to Tem/Teffe cells, while in non-responders, STMN2+ TAM were nearer to CD8+ Tex cells (**Figure 5E**). A heatmap depicting the k-distance to STMN2+ TAM in each section corroborated these findings (**Figure 5F**). Additionally, we applied the RRA technique to generate an extensive rank of the k-distance to STMN2+ TAM, showing that in responders, STMN2+ TAM were situated closer to Tem/Teffe cells, whereas in non-responders, STMN2+ TAM were positioned nearer to CD8+ Tex cells (**Figure 5G**). Overall, the spatially resolved stRNA-seq analysis revealed that the proximity between STMN2+ TAM and CD8+ Tex cells may significantly influence the response to immunotherapy in tumor patients.

### TAM heterogeneity in NB

**Figure 6:**
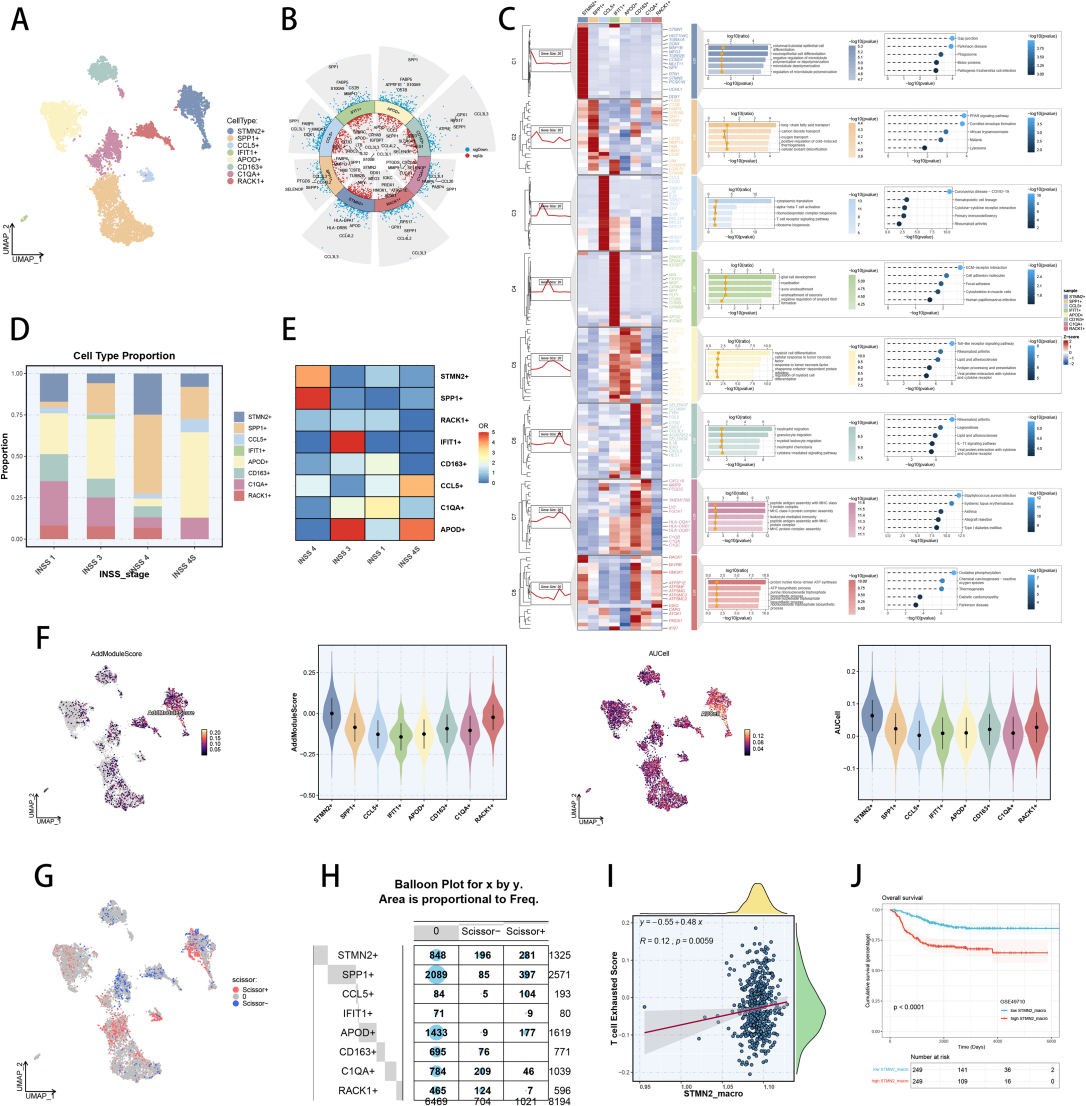
TAM heterogeneity and identification of TEX-related TAM subsets in NB scRNA-seq cohort. **(A)** Color-coded UMAP plot of TAM subtypes in NB scRNA-seq cohort. **(B)** Marker genes of each TAM subtype. **(C)** Functional enrichment of each TAM subsets via Biological Process of GO terms and KEGG database. **(D)** The cell proportion of each TAM subtype in every INSS stage. **(D)** The OR values of each TAM subtype in every INSS stage. **(F)** The proportion of responder cells and non-responder cells in each major cell type. F) Color-coded UMAP plot of TEX enrichment scores in TAMs, and comparison of TEX enrichment scores among TAM subsets by AddModuleScore and AUCell algorithm. **(G)** The cell distribution of scissor+ and scissor- cells in TAMs. **(H)** The compositions of scissor+ and scissor- cells in different TAM subsets. **(I)** Spearman correlation of TEX scores with STMN2+ TAM risk scores in GSE49710 cohort. **(J)** Survival analysis of different groups divided by STMN2+ TAM risk score in GSE49710 cohort.

Next, we researched the heterogeneity of TAMs within NB specific context and ultimately defined eight TAM sub types (**Figure 6A**). Based on literature review, we successfully annotated various TAM phenotypes which notably expressed hub genes of SPP1, C1QA, CCL5, IFIT1 and STMN2 (**Figure 6A**, **Supplementary Figures S6A–C**) (47, 77). We depicted their typical marker genes of each TAM subtype in circle plot (**Figure 6B**). Functional enrichment analysis revealed the diverse biological roles of these TAM subtypes in TIME (**Figure 6C**). It was observed that the prevalence of STMN2+ TAM and SPP1+ TAM were abundant in stage 4 NB, indicating a potential influence of these subpopulations in malignant behaviors (**Figure 6D**). Additionally, OR analysis of STMN2+ TAM and SPP1+ TAM revealed their enrich in stage 4 NB (**Figure 6E**). Hence, we utilized scRNA-seq scoring method within every TAM phenotype based on TEX markers. The results indicated that STMN2+ TAM had the top values, suggesting their close relationship with TEX (**Figure 6F**). We also utilized “scissor” algorithm to identify TAM subpopulations associated with poor prognosis (**Figure 6G**). And we found that scissor+ cells were abundant in APOD+ TAM, CCL5+ TAM, STMN2+ TAM and SPP1+ TAM (**Figure 6H**), revealing potential malignant roles of these subpopulations. Subsequently, we calculated the enrichment scores of STMN2 TAM signature and TEX signature via ssGSEA technique in RNA-seq cohort of NB, indicating their potentially tight relationship in NB (**Figure 6I**). Additionally, survival analysis revealed that NB patients with higher STMN2+ TAM scores would suffer poorer prognosis, demonstrating the unfavorable prognosis role of STMN2+ TAM (**Figure 6J**). Our analysis demonstrated that STMN2+ TAM possibly influenced both immunotherapy responsiveness and patient prognosis in pan-cancer, as well as NB specific context.

### Identification of TEX-related NB tumor cells

**Figure 7:**
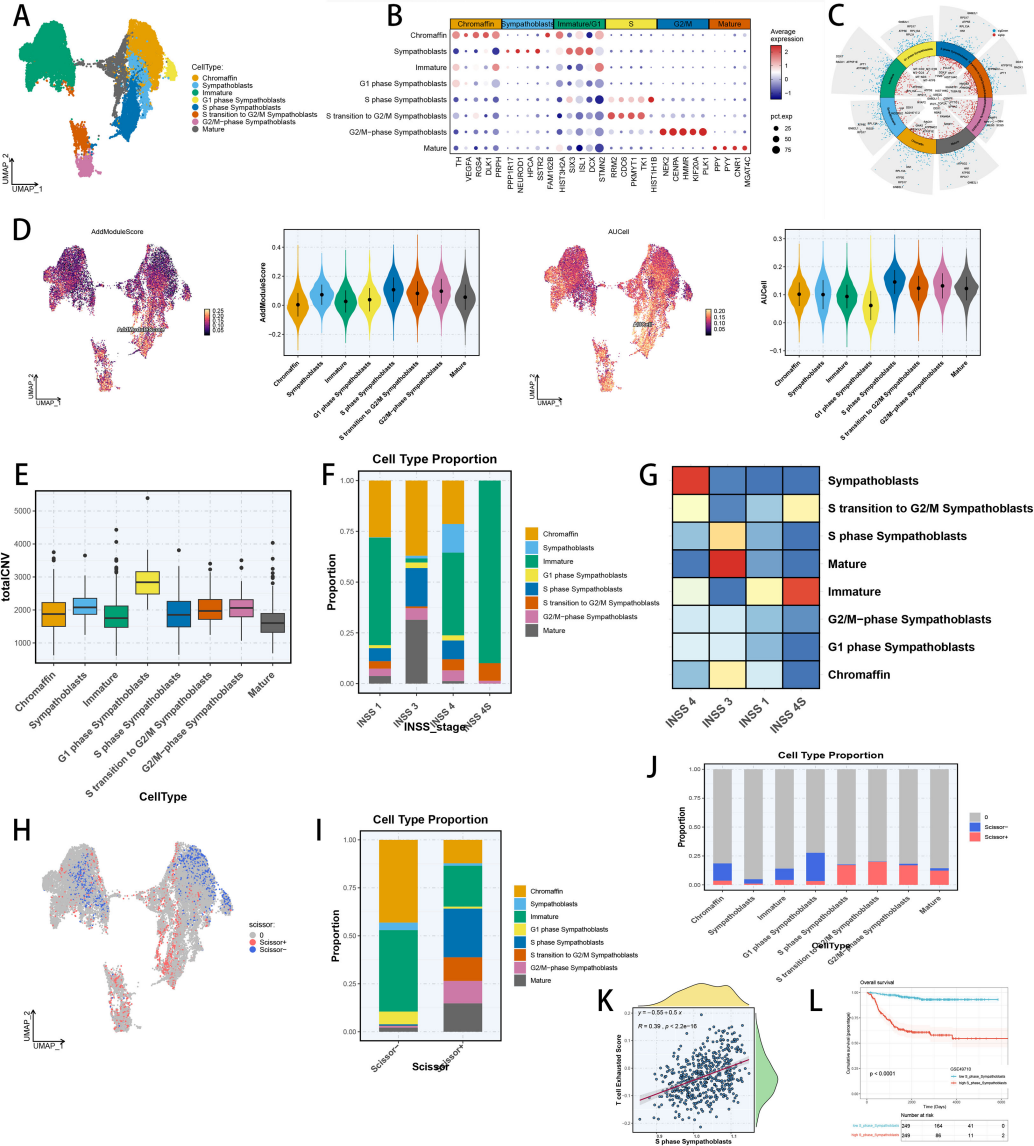
The heterogeneity of neuroendocrine cells and identification of TEX-related neuroendocrine cell subsets in NB scRNA-seq cohort. **(A)** Color-coded UMAP plot of neuroendocrine cell subtypes in NB scRNA-seq cohort. **(B, C)** Canonical markers and typical marker genes of each neuroendocrine cell subtype. **(D)** Color-coded UMAP plot of TEX enrichment scores in neuroendocrine cells, and comparison of TEX enrichment scores among neuroendocrine cell subsets by AddModuleScore and AUCell algorithm. **(E)** The totalCNV of each neuroendocrine cell subset by inferCNV analysis. **(F)** The cell proportion of each neuroendocrine cell subtype in every INSS stage. **(G)** The OR values of each neuroendocrine cell subtype in every INSS stage. **(H)** The cell distribution of scissor+ and scissor- cells in neuroendocrine cells. **(I)** The cellular proportions of neuroendocrine cell subpopulations between scissor+ and scissor- cells. **(J)** The compositions of scissor+ and scissor- cells in different neuroendocrine cell subsets. **(K)** Spearman correlation of TEX scores with S phase sympathoblasts risk scores in GSE49710 cohort. **(L)** Survival analysis of different groups divided by S phase sympathoblasts risk score in GSE49710 cohort.

To assess the TEX-related NB tumor cells with malignant behaviors, we then analyzed the neuroendocrine cells in NB. We used UMAP to illustrated the various neuroendocrine subpopulations (**Figure 7A**), based on canonical markers from literature review (78) (**Figure 7B**) and cell cycle scoring in single-cell level (**Supplementary Figure S6D**). Meanwhile, the marker genes of each neuroendocrine subtype validated our annotations (**Figure 7C**). Moreover, functional enrichment of each neuroendocrine subpopulations further depicted the biological behaviors of various subtypes (**Supplementary Figure S6E**). Besides, we utilized scRNA-seq scoring algorithms to discover the TEX-related NB tumor cells, identifying that S phase sympathoblasts were correlated with TEX (**Figure 7D**). Additionally, “infercnv” technique was utilized to explore the CNV counts in single-cell levels among neuroendocrine subpopulations, revealing the potential malignant role of S phase sympathoblasts (**Figure 7E**). It was observed that the prevalence of S phase sympathoblasts, mature neuroendocrine cells and sympathoblasts were significantly elevated in stage 3 and 4 NB (**Figure 7F**). And OR values of S phase sympathoblasts and sympathoblasts were high in stage 3 and 4 NB (**Figure 7G**). Next, “scissor” technique was used to define the cell subset associated with poor prognosis (**Figure 7H**). We discovered that S phase sympathoblasts occupied a huge proportion of scissor+ cells (**Figure 7I**), while most of S phase sympathoblasts was scissor+ cells (**Figure 7J**), indicating its malignant behaviors. Subsequently, we computed the enrich scores of S phase sympathoblasts and TEX through ssGSEA technique in RNA-seq dataset of NB, revealing their potentially tight association in NB (**Figure 7K**). Additionally, survival analysis demonstrated that NB patients with higher S phase sympathoblasts scores would suffer poorer prognosis, illustrating the unfavorable prognosis role of S phase sympathoblasts (**Figure 7L**). Our resulted advised that S phase sympathoblasts were tightly related to TEX and would hugely affect patient prognosis in NB.

### Assessment of TEX-related TIME in NB

**Figure 8:**
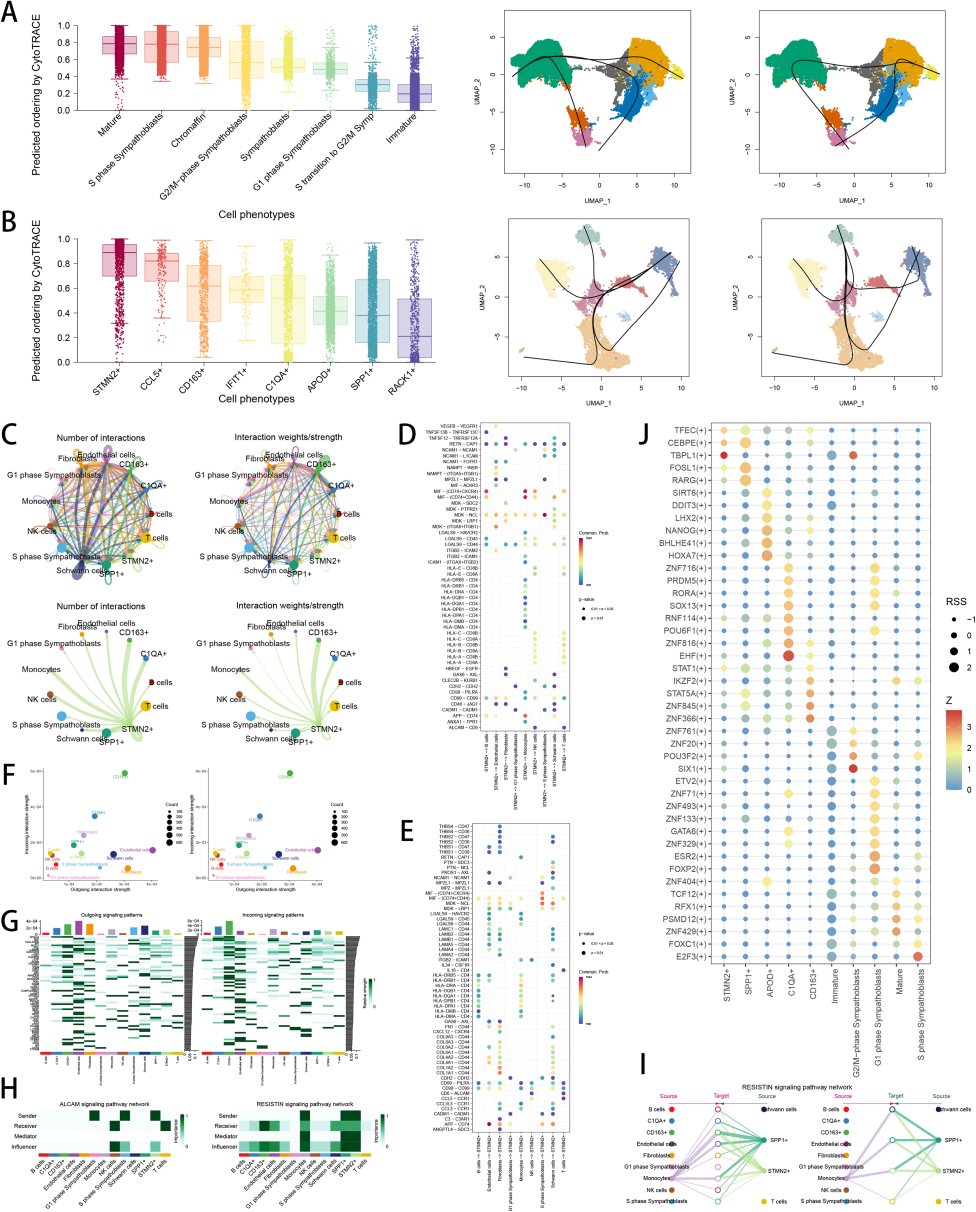
Assessment of TEX-related tumor immune microenvironment in NB via scRNA-seq analysis. **(A)** CytoTRACE scores were visualized with boxplot, and slingshot were used to perform pseudotime analyses to infer cellular differentiation states in neuroendocrine cell subpopulations. **(B)** CytoTRACE scores were visualized with boxplot, and slingshot were used to perform pseudotime analyses to infer cellular differentiation states in TAM subpopulations. **(C)** Analysis of cell–cell communication intensity and quantity between cell subtypes through Cellchat method. **(D, E)** Receptor-ligand pairs among STMN2+ TAM and various cell subtypes. **(F)** The relationship between differential outgoing interactions and incoming interaction strength for different cell subtypes. **(G)** Heatmap showing incoming and outgoing interactions among different cell subtypes. **(H)** The sender, receiver, mediator and influencer of ALCAM and RESISTIN signaling pathways in different cell subclusters. **(I)** The source and target of RESISTIN signaling pathway in different cell subclusters. **(J)** Comparison of transcription factor activities among cell subclusters.

To gather deeper insights into TEX-related TIME in NB, we utilized pseudotime analysis to explore cellular differentiation trajectory in NB. We demonstrated the overall landscapes of cellular differentiation in neuroendocrine subpopulations (**Figure 8A**) and TAM subpopulations (**Figure 8B**) via “CytoTrace” and “Slingshot” analysis. The TEX-related subsets, STMN2+ TAM, as well as S phase sympathoblasts, both scored highly in stemness. Meanwhile, “Cellchat” analysis was used to illustrate intercellular communications among neuroendocrine subpopulations and TAM subpopulations (**Figure 8C**), with STMN2+ TAM displaying strong strength and weights in cell communication. Then, we explored specific interactions of LRIs among STMN2+ TAM and various cell subsets within TIME (**Figures 8D, E**), shedding lights on potential communication targets in TEX-related subsets. Specifically, we found that TAMs had higher incoming communication signals in responders and non-responders (**Figure 8F**), whereas CD163+ TAM and endothelial cells were mostly engaged in responders and non-responders (**Figure 8F**). Additionally, we found that cellular interactions of ingoing and outgoing patterns were significantly diverse among those cell populations (**Figure 8G**). We then explored the ALCAM and RESISTIN signaling pattern networks in TIME, revealing STMN2+ TAM importantly engaged in those networks (**Figures 8H, I**). Next, we analyzed the TF regulon network in TEX-related TIME of NB, demonstrating that STMN2+ TAM were regulated by TBPL1 and CEBPE regulon (**Figure 8J**). In summary, we depicted the comprehensive landscapes of differential trajectory, cellular interplay and TF networks in TEX-related TIME, indicating potential biological meaning underlying TEX-regulated TIME in NB.

### Establishment and verification of pan-cancer STMN2.SIG

**Figure 9:**
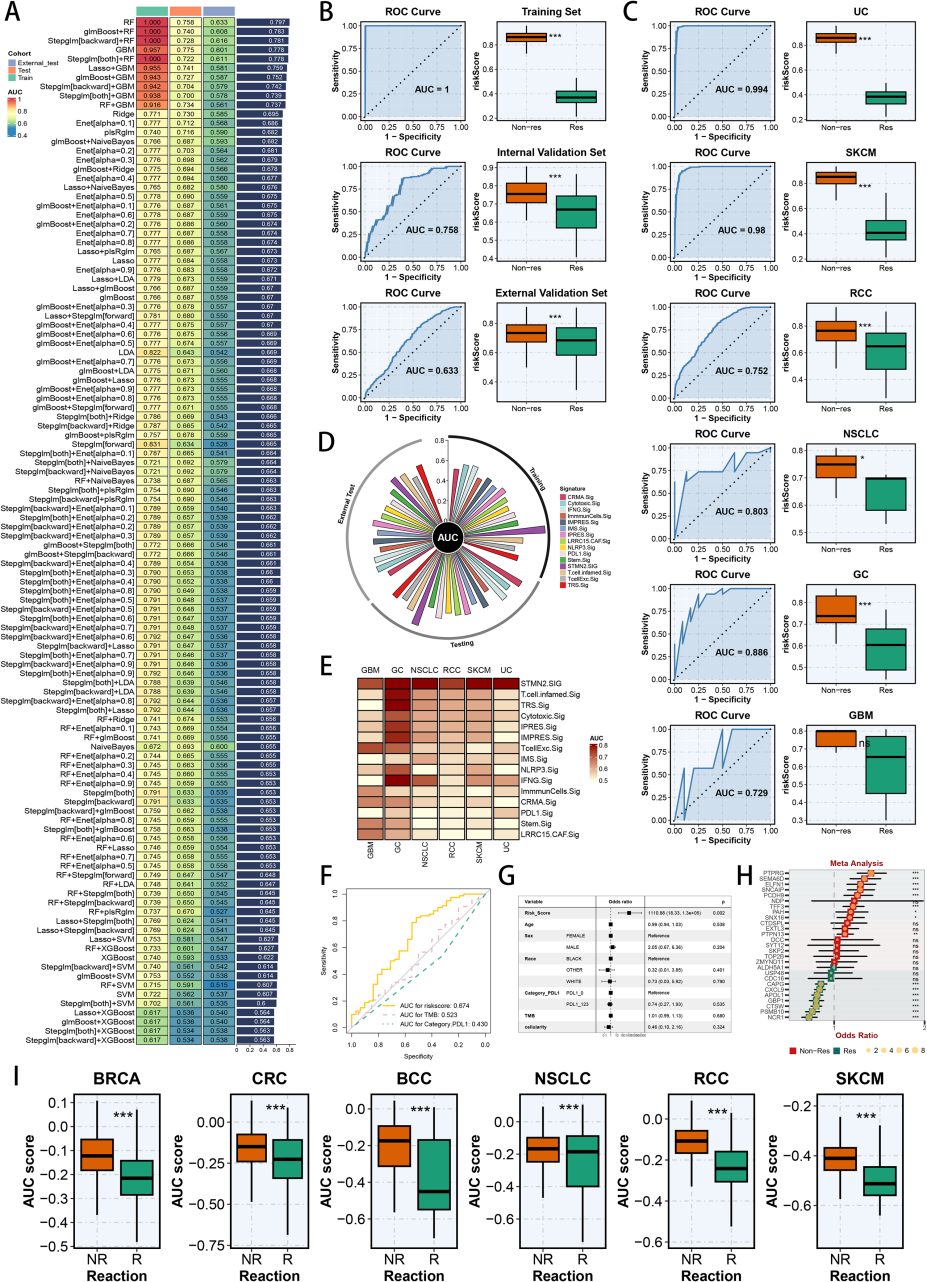
Development and validation of pan-cancer STMN2.SIG to predict immunotherapy response. **(A)** Construction of pan-cancer STMN2.SIG using various machine learning combinations, with values in the heatmap representing the AUC of corresponding models for predicting immunotherapy response; the bar graph on the right shows the average AUC across multiple datasets. **(B)** ROC curves of pan-cancer STMN2.SIG to predict immunotherapy response (left); as well as the distribution of pan-cancer STMN2.SIG risk scores between responders and non-responders (right), in the training, internal validation, and external validation cohorts. **(C)** ROC curves of the pan-cancer STMN2.SIG to predict immunotherapy response (left); as well as the distribution of pan-cancer STMN2.SIG risk scores between responders and non-responders (right), in the UC, SKCM, RCC, NSCLC, GC and GBM cohorts. **(D)** Comparison of the AUCs among pan-cancer STMN2.SIG and other published signatures in the training, internal validation, and external validation cohorts (Bar of AUC > 0.8 was not plotted). **(E)** Comparison of the AUCs among pan-cancer STMN2.SIG and other published signatures in the UC, SKCM, RCC, NSCLC, GC and GBM cohorts. **(F)** Comparing pan-cancer STMN2.SIG risk scores with TMB and PDL-1 based on ROC Curves in the IMmotion150 cohort. **(G)** Multivariate logistic regression analysis in the IMmotion150 cohort. **(H)** Meta-analysis of odds ratio of model genes from pan-cancer STMN2.SIG in the training, internal validation, and external validation cohorts. **(I)** The different pan-cancer STMN2.SIG risk score between immunotherapy responders and non-responders across six cancer types. *p < 0.05; **p < 0.01; ***p < 0.001; ****p < 0.0001.

Given the highlighted potential of STMN2+ TAM to influence TIME in scRNA-seq analysis, we proceeded to analyze their marker genes in RNA-seq data for practical application and clinical validation. To better understand the predictive potential of these genes, we gathered RNA-seq and clinical data from 16 immunotherapy related cohorts, covering diverse tumors. As outlined earlier, these cohorts were partitioned into the train, internal test, and external test cohorts, to utilize the STMN2+ TAM marker genes for model development. PCA plots visualized well adjustment of batch effects before and after batch correction (**Supplementary Figure S7A**). By employing 113 predictive ML approaches and a LOOCV system, we refined a highly significant model and benchmarked it against existing in immunotherapy related signatures (**Figure 9A**). The most potent signature was created using RF for feature selection and signature building, yielding the highest average AUC (0.797) across the three cohorts (**Figure 9A**). The AUC of the optimal signature after nested cross-validation was depicted for the train (AUC = 1.00, 95% CI: 1.00−1.00), internal test (AUC = 0.76, 95% CI: 0.67−0.84) and external test (AUC = 0.63, 95% CI: 0.59−0.68) cohorts, revealing that non-responders exhibited significantly higher risk scores (**Figure 9B**). Additionally, in the combined datasets of every cancer type, the pan-cancer STMN2.SIG demonstrated superior AUC values, with non-responders consistently showing higher risk scores (**Figure 9C**). Relative to existing immunotherapy related signatures, the pan-cancer STMN2.SIG achieved markedly higher AUC values in RCC patients, highlighting its superior predictive capacity for immunotherapy response in RCC patients (**Supplementary Figure S7B**). Furthermore, the pan-cancer STMN2.SIG exhibited enhanced predictive capabilities across the training, internal validation, and external validation datasets (**Figure 9D**), as well as in the aggregated datasets of different cancer types (**Figure 9E**). We subsequently conducted the confusion matrix to illustrate the high precision of pan-cancer STMN2.SIG (**Supplementary Figure S7C**). Utilizing the extensive clinical data from the IMmotion150, IMmotion151, Mariathasan, Braun, and Riaz cohorts, we generated ROC curves to evaluate pan-cancer STMN2.SIG’s predictive performance in conjunction with PD-L1 expression and TMB, which consistently outperformed both PD-L1 and TMB across multiple cohorts (**Figure 9F**; **Supplementary Figure S7D**). Further, multivariate logistic regression analysis confirmed the risk score as an independent predictor of clinical response to immunotherapy (all p < 0.05) (**Figure 9G**; **Supplementary Figure S7E**). Within the Braun cohort, we evaluated the AUC of pan-cancer STMN2.SIG, several clinical variables, and the nomogram model, emphasizing pan-cancer STMN2.SIG’s superior predictive power (**Supplementary Figure S7F**). Calibration curves revealed a strong concordance between the signature’s predicted and observed probabilities (**Supplementary Figure S7G**), revealing the well consistency, accuracy and stability of pan-cancer STMN2.SIG. DCA curves in the Braun cohort further highlighted the net clinical advantage of pan-cancer STMN2.SIG, which outperformed other clinical predictors (**Supplementary Figure S7H**). We subsequently employed the nomogram to integrate several clinical factors along with pan-cancer STMN2.SIG, facilitating clinical decision-making (**Supplementary Figure S7I**). Furthermore, using univariate logistic analysis to appraise the prediction value of model genes across three cohorts, we depicted the odds ratios of each model gene through meta-analysis. This analysis identified the risky and protective model genes associated with immunotherapy non-response (**Figure 9H**). Additionally, the risk score is consistently higher in non-responders compared to responders across all cancer types, suggesting that STMN2+ TAM may significantly contribute to the failure of immunotherapy (**Figure 9I**).

### Development and validation of NB-specific STMN2.SIG

**Figure 10:**
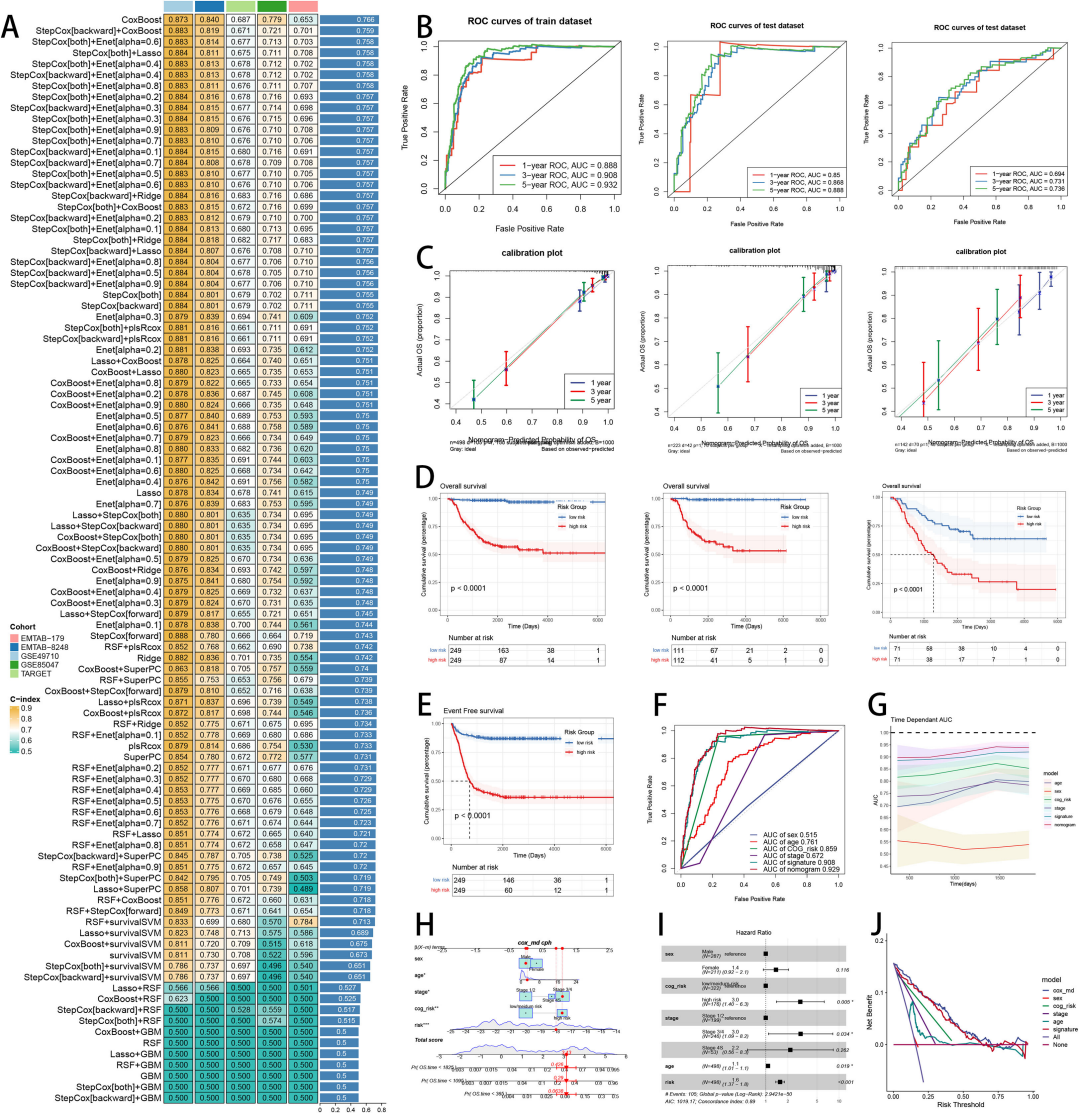
Development and verification of NB-specific STMN2.SIG to predict prognosis in NB. **(A)** A total of 101 kinds of prognostic models via a leave-one-out cross-validation framework and further calculated the C-index of each model. **(B)** ROC curves of 1-, 3- and 5-year OS of NB-specific STMN2.SIG in GSE49710, E-MTAB-8248, and TARGET cohort. **(C)** 1-, 3- and 5-year calibration curves of NB-specific STMN2.SIG in GSE49710, E-MTAB-8248, and TARGET cohort. **(D)** Kaplan-Meier survival curves of OS for high-risk and low-risk groups of NB patients in GSE49710, E-MTAB-8248, and TARGET cohort. **(E)** Kaplan-Meier survival curves of EFS for high-risk and low-risk groups of NB patients in GSE49710 cohort. **(F)** AUC values of 3-year OS of NB-specific STMN2.SIG, the cox regression model and clinical variables in GSE49710 cohort. **(G)** Time dependent ROC curves of NB-specific STMN2.SIG, the cox regression model and clinical variables in GSE49710 cohort. **(H)** The nomogram illustration of NB-specific STMN2.SIG in GSE49710 cohort. **(I)** Forest plot visualized the outcome of multivariate Cox regression analysis involving NB-specific STMN2.SIG and clinical variables. **(J)** DCA curves of NB-specific STMN2.SIG, the cox regression model and clinical variables in GSE49710 cohort.

Having validated the pan-cancer STMN2.SIG’s utility in predicting immunotherapy response, we next investigated its broader applicability in pediatric cancer prognosis, which is our research focus for pediatric surgeons, as frontline immunotherapy is not standard in NB. To explore the outperforming predictive value of STMN2.SIG in prognosis forecast for NB, we sought to develop and verify a potent NB-specific STMN2.SIG. We then conducted 101 prognosis ML combinations with LOOCV system to sift through the best ML framework to develop the signature (**Figure 10A**). Ultimately, the most effective prognosis ML combination was developed by CoxBoost in feature selection and signature development with the top average C-index (0.766) in five cohorts (**Figure 10A**). ROC curves in 1- (AUC: 0.888, 95% CI: 0.819-0.945), 3- (AUC: 0.908, 95% CI: 0.869-0.944) and 5-year (AUC: 0.932, 95% CI: 0.901-0.963) OS demonstrated superior specificity of NB-specific STMN2.SIG in the train cohort, as well as internal and external validation cohorts (**Figure 10B**). Calibration curves showed the well consistency, accuracy and stability of NB-specific STMN2.SIG in three cohorts (**Figure 10C**), indicating that the model’s possibility predictions were consistent and well-calibrated. Survival analysis depicted that the low-risk patients had a better OS (**Figure 10D**) and EFS (**Figure 10E**) than the high-risk patients in NB datasets. AUC values of 3-year OS displayed that NB-specific STMN2.SIG and cox regression model were more discriminative to forecast prognosis than other clinical indicators (**Figure 10F**). Time dependent AUC values verified that NB-specific STMN2.SIG and cox regression model acted better than several clinical indicators in discriminative capability (**Figure 10G**). Nomogram of the cox regression model covering NB-specific STMN2.SIG was illustrated (**Figure 10H**). Multivariate Cox regression analysis validated that NB-specific STMN2.SIG risk score was an independently prognostic predicator in GSE49710 cohort (P < 0.001) (**Figure 10I**). DCA curves indicted that predicting prognosis with NB-specific STMN2.SIG would provide net clinical benefits (**Figure 10J**). In summary, those indicators of model assessment comprehensively demonstrated that NB-specific STMN2.SIG showed precision and robustness in model performance.

### Model comparison

**Figure 11:**
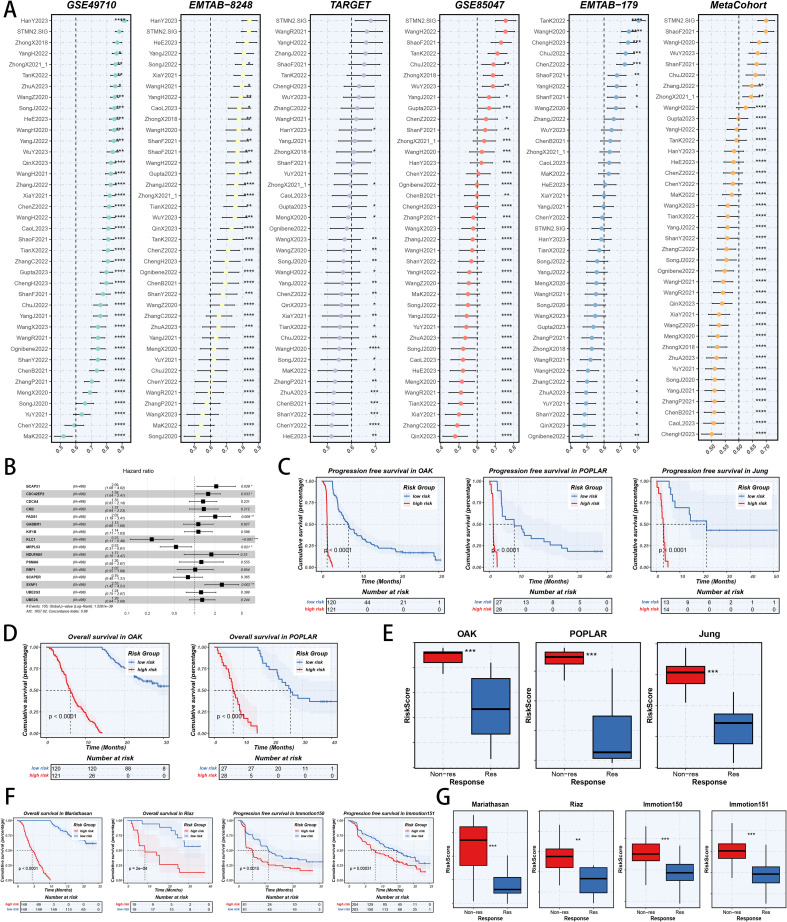
Model comparisons and model performances in predicting immunotherapy related prognosis. **(A)** C-index comparison analysis between NB-specific STMN2.SIG and published signatures in GSE49710, E-MTAB-8248, TARGET, GSE85047, E-MTAB-179 and the meta-cohort. *p < 0.05; **p < 0.01; ***p < 0.001; ****p < 0.0001. **(B)** The univariate cox regression analysis of model variables of NB-specific STMN2.SIG. **(C)** Kaplan-Meier survival curves of PFS for high-risk and low-risk groups in three immunotherapy treated cohorts. **(D)** Kaplan-Meier survival curves of OS for high-risk and low-risk groups in two immunotherapy treated cohorts. **(E)** Comparison of risk scores calculated by NB-specific STMN2.SIG in responders and non-responders in three immunotherapy treated cohorts. **(F)** Kaplan-Meier survival curves of OS or PFS for high-risk and low-risk groups in IMmotion150, IMmotion151, Mariathasan, and Riaz cohorts. **(G)** Comparison of risk scores calculated by NB-specific STMN2.SIG in responders and non-responders in IMmotion150, IMmotion151, Mariathasan, and Riaz cohorts.

To test the outperforming prognosis prediction capability of STMN2.SIG in NB specific context, we collected model gene coefficients of various previously published NB prognosis models. Subsequently, we analyzed C-index of those prognostic models with STMN2.SIG in NB RNA-seq cohorts. Finally, we demonstrated that the STMN2.SIG performed better than most of those models in NB datasets in prognosis prediction (**Figure 11A**), which indicated that STMN2.SIG acted as a powerful NB prognostic model. Then, we illustrated the hazard ratio of STMN2.SIG model genes selected by CoxBoost algorithm, calculated by univariate cox regression analysis (**Figure 11B**). For immunotherapy prognosis prediction, we performed survival analysis in three immunotherapy related cohorts of NSCLC, and discovered that patients with high risk points would suffer worse PFS (**Figure 11C**) and OS (**Figure 11D**) than patients with low risk points. Moreover, responders of immunotherapy would have lower risk scores than non-responders of immunotherapy, proving the predictive capability of STMN2.SIG in immunotherapy response (**Figure 11E**). To further assess the pan-cancer applicability of STMN2.SIG, we validated its ability of risk-stratification in IMmotion150, IMmotion151, Mariathasan, and Riaz cohorts. The signature consistently stratified patients into high-risk and low-risk groups with significant differences in survival outcomes (**Figure 11F**), underscoring its robust predictive power beyond NSCLC. Immunotherapy responders exhibited significantly lower STMN2.SIG risk scores than non-responders, validating the signature’s predictive power for treatment response (**Figure 11G**).

### Expression validations and functional experiments

**Figure 12:**
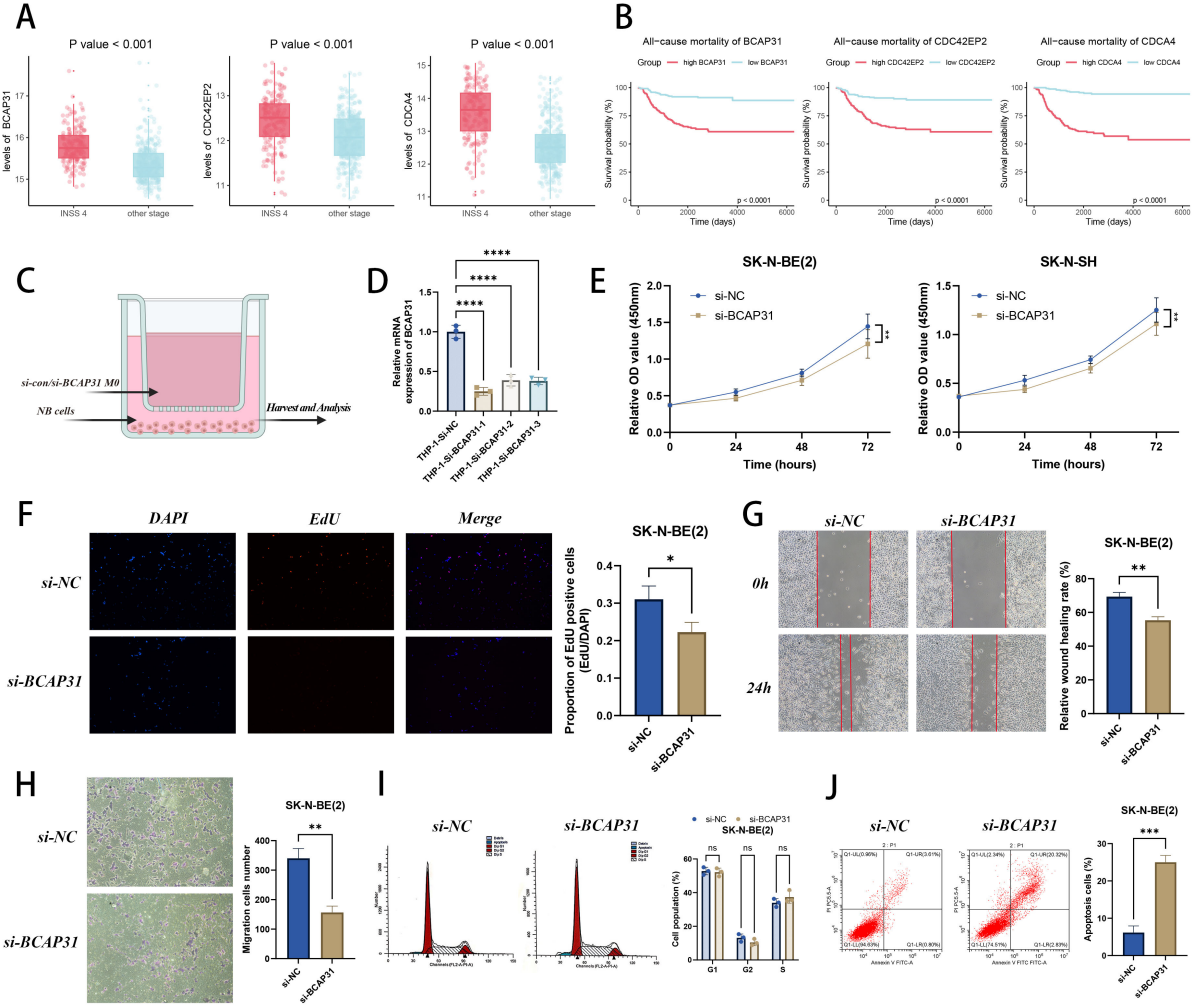
Experimental validation of hub gene BCAP31 in macrophages. **(A)** Different expressions of three hub genes (BCAP31, CDC42EP2 and CDCA4) in INSS stage 4 tumors and other INSS stages tumors in GSE49710 cohort. **(B)** Prognosis value of three hub genes (BCAP31, CDC42EP2 and CDCA4) illustrated by K-M curves in GSE49710 cohort. **(C)** Illustration of co-culture system. **(D)** qRT-PCR analysis of BCAP31expression in TMP-1 cells after transfection with siRNAs. **(E)** CCK8 assay of SK-N-SH (nonamplified MYCN) and SK-N-BE(2) (amplified MYCN) cell line co-cultured with M0 macrophages knock down with/without BCAP31. **(F–H)** Silencing of BCAP31 in M0 macrophages suppressed NB cell proliferation, invasion, and migration after co-culture. **(I, J)** Silencing of BCAP31 in M0 macrophages did not influence NB cell cycle but promoted apoptosis in NB cells. *, **, ***, and **** indicate a significance level of 0.05, 0.01, 0.001, and 0.0001, respectively.

To further explain the underlying functional mechanism of STMN2.SIG, we successfully discovered hub genes of STMN2.SIG (BCAP31, CDC42EP2 and CDCA4). In GSE49710 cohort, they expressed significantly more in stage 4 NB (**Figure 12A**) and were unfavorable prognosis marker in NB (**Figure 12B**). Building on these findings, we embarked on an investigation into the influence of BCAP31 on TAM in TIME. Through co-culture experiments (**Figure 12C**), we explored how BCAP31 on TAM might affect NB tumor cells, employing NB tumor cells co-cultured with THP-1-derived macrophages where BCAP31 was specifically knocked down. We obtained three siRNA to silence BCAP31 in TAM and we used siRNA-1 in the following function experiments due to its best effectiveness (**Figure 12D**). Loss-of-function experiments displayed that silence of BCAP31 on TAM significantly inhibited the proliferation, migration and invasion capability of NB cell line with CCK-8 assay (**Figure 12E**), EdU assay (**Figure 12F**), wound healing assay (**Figure 12G**), and transwell assay (**Figure 12H**), respectively. Moreover, flow cytometry analysis demonstrated that knockdown of BCAP31 had no significant influence on cell cycle of NB cell line (**Figure 12I**) but promoted apoptosis of NB cell line (**Figure 12J**). In summary, we discovered that silencing BCAP31 in TAM could significantly inhibit the malignant behaviors of NB tumor cells in the co-culture system.

### BCAP31 on TAM affects immune effector functions of CD8+ T cells

**Figure 13:**
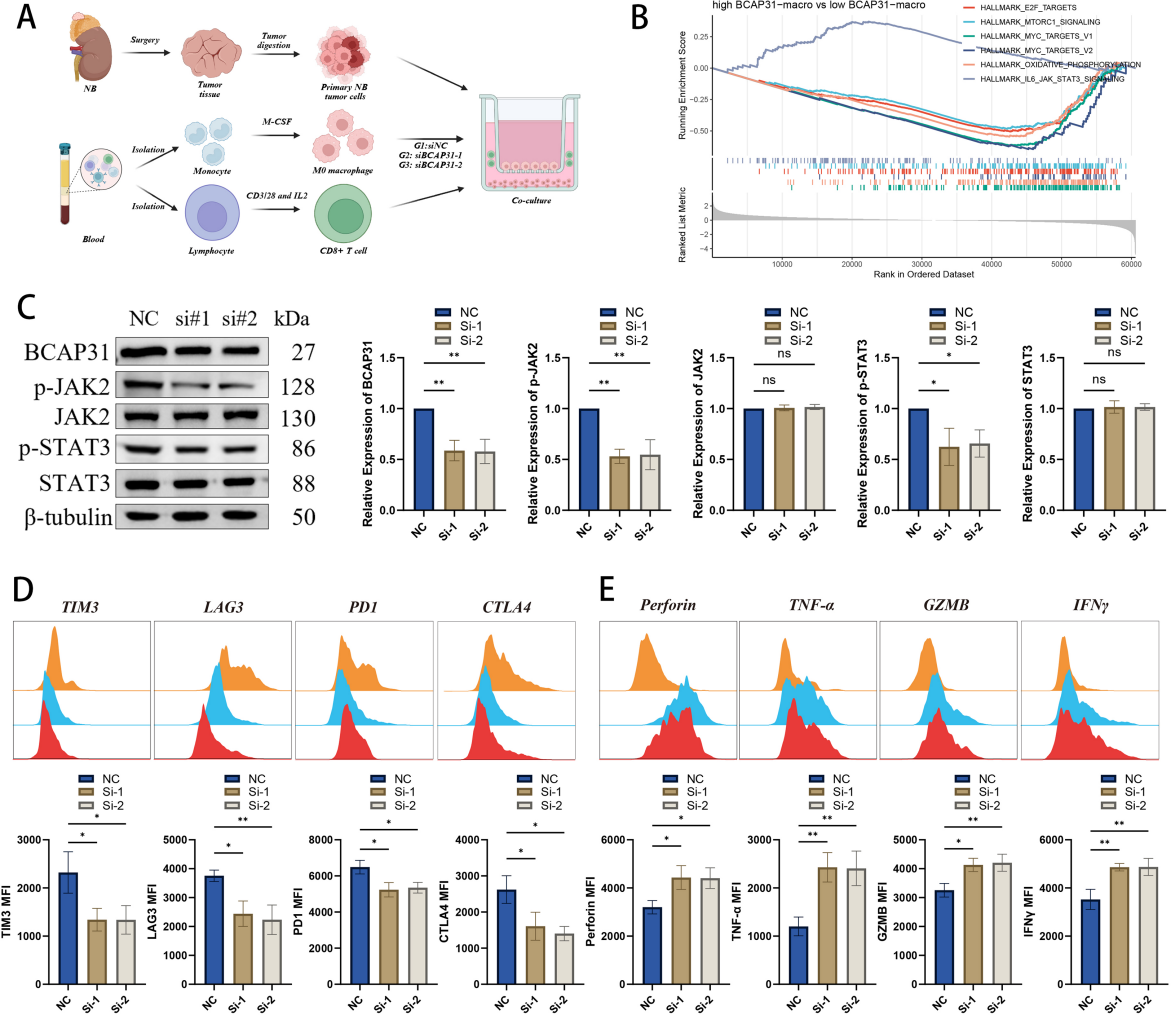
Down-regulating BCAP31 in TAMs promotes intra-tumoral CD8+ T cells function and drives them toward an effector state. **(A)** Workflow of the co-culture system with primary TAMs (NC, siBCAP31-1 and siBCAP31-2), primary NB tumor cells, and CD8+ T cells. **(B)** GSEA analysis in GSE49710 cohort (high BCAP31-macro vs low BCAP31-macro). **(C)** Representative western blot of BCAP31, p-JAK2, JAK2, p-STAT3, STAT3 and β-tubulin protein expression levels. **(D)** Flow cytometry analysis of TIM-3, PD-1, LAG3, and CTLA4 in CD8+ T cells isolated from the co-culture system. **(E)** Flow cytometry analysis of GZMB, IFN-γ, Perforin, and TNF-α in CD8+ T cells isolated from the co-culture system. *p < 0.05; **p < 0.01; ***p < 0.001; ****p < 0.0001.

**Figure 14:**
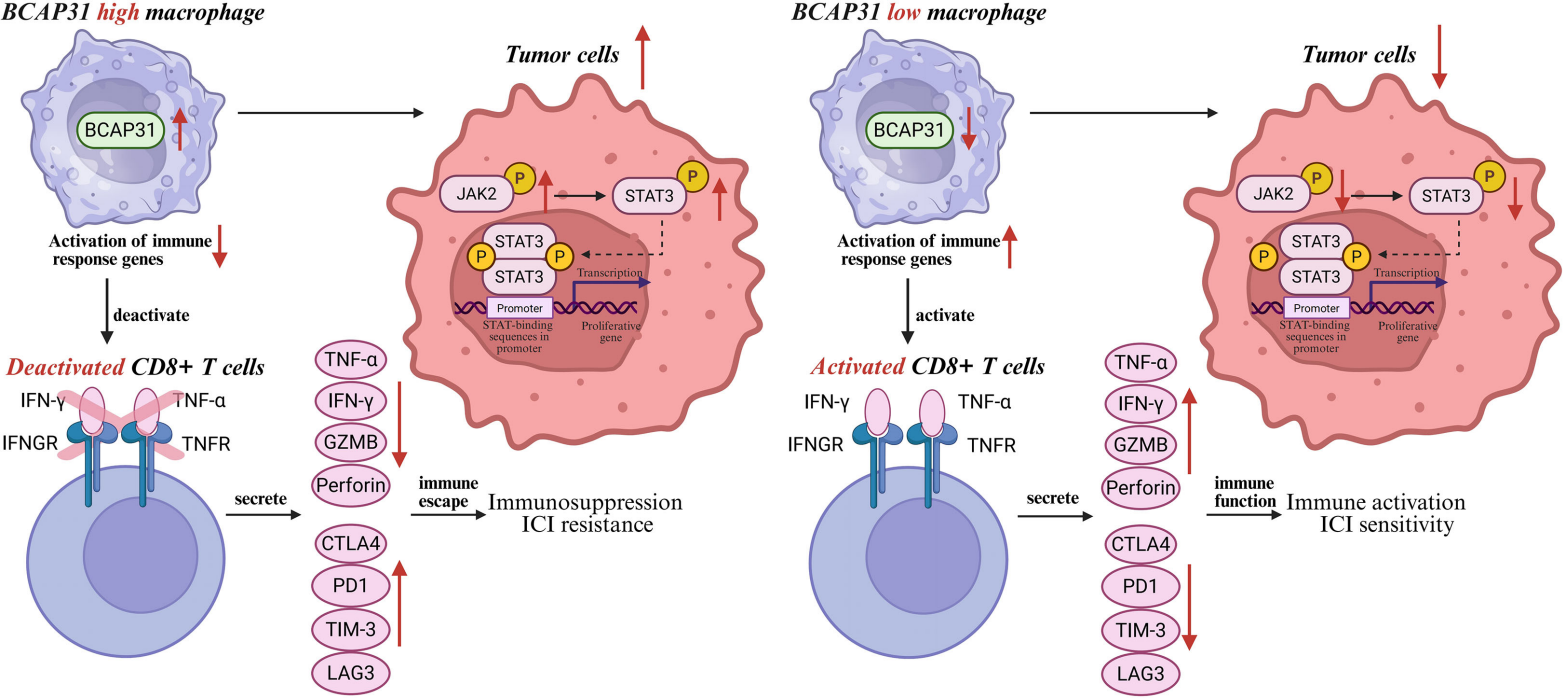
Schematic model depicting the key findings of this study. Silencing BCAP31 in TAMs could inactivate JAK2-STAT3 signaling pathway in tumor cells, as well as caused the productions of pro-immune cytokines and resulting in activated CD8+ T cells. The activated CD8+ T cells would secrete more cytokines and release less exhausted molecules, which finally led to immune activation and immunotherapy sensitivity.

From the aforementioned results, it appears that BCAP31 on TAM have a negative impact on the prognosis of NB cases. To explore whether reduced BCAP31 expression could enhance the cytotoxicity of CD8+ T cells *in vitro*, we extracted primary tumor cells from tumor samples of HLA-A2+ NB patients. We also isolated macrophages and CD8+ T cells from the patients’ PBMCs. Primary macrophages were divided into three groups: NC, siBCAP31-1, and siBCAP31-2. To further investigate the role of BCAP31 in regulating immune effector function of TAM, an indirect co-culture system was established involving primary macrophages (NC, siBCAP31-1, and siBCAP31-2), primary NB cells, and CD8+ T cells (**Figure 13A**). To elucidate the mechanisms underlying the effector function, we conducted GSEA analysis in GSE49710 cohort. The analysis revealed that cases with high BCAP31 expression on TAM exhibited elevated enrichment of “IL6-JAK-STAT3” pathway compared to those with low BCAP31 expression on TAM (**Figure 13B**). The successful knockdown of BCAP31 in TAM was confirmed by WB (**Figure 13C**), which demonstrated that silencing BCAP31 in TAM significantly down-regulated the protein levels of p-JAK2 and p-STAT3, while protein levels of JAK2 and STAT3 were not significantly altered (**Figure 13C**). We subsequently extracted CD8+ T cells from the co-culture system and employed flow cytometry to demonstrate that the effector function of CD8+ T cells was enhanced following BCAP31 silencing of macrophages, while exhaustion markers were diminished (**Figures 13D, E**). To sum up, our findings indicate that silencing BCAP31 in TAM can remarkably inactivate the JAK2-STAT3 signaling pathway in tumor cells and enhance the effector function of CD8+ T cells, serving as a promising therapeutic strategy in NB. Collectively, we hypothesized that silencing BCAP31 in TAM could inactivate the JAK2-STAT3 signaling pathway in tumor cells, and leading to enhanced levels of pro-immune cytokines, consequently, activated CD8+ T cells. These activated CD8+ T cells would secrete more cytokines and express lower levels of exhaustion markers, ultimately contributing to immune activation for killing NB cells (**Figure 14**).

## Discussion

Immunotherapy has now become primary and innovative adjuvant treatments for most early-stage and advanced cancers. The prior pan-cancer study has successfully revealed a link between TAMs and immunotherapy efficacy in various cancers, with CD8+ T cells frequently found in TAM-rich areas of TIME (79). This suggested that TAMs might dampen antitumor T-cell immunity in solid tumors, correlating with poor prognosis and treatment failure. Prior research has largely concentrated on elucidating the connection between TAMs and clinical traits (80). For instance, researchers devised a TAM-associated signature capable of forecasting outcomes, leveraging RNA-seq data from breast cancer. This signature’s associated risk score exhibits a strong correlation with both the pathological attributes of tumors and the IC50 values of targeted therapies (81). Recently, researchers identified a TAM-associated gene signature and demonstrated its potential link to cytotoxic T lymphocyte (CTL) exhaustion across various malignancies, yet they did not elucidate its function in immunotherapy (82). Moreover, numerous studies have shown that TAM-related genes or gene signatures can influence TIME and immunotherapy outcomes (83). In recent times, the application of ML in the medical field for predictive purposes has increasingly gained traction, encompassing areas such as survival prognosis, metastatic potential, and therapeutic efficacy, with promising outcomes (84, 85). However, this method has not been extensively utilized in the context of macrophages, particularly for forecasting responses to immunotherapy.

In this research, we examined TEX-associated TAM subsets in immunotherapy responders and non-responders at a single cell resolution. Subsequently, we constructed a predictive model (pan-cancer STMN2.SIG) for immunotherapy response by leveraging diverse ML techniques and integrating comprehensive scRNA-seq and bulk transcriptomic data, thereby enhancing prediction accuracy and showcasing innovation. Totally 113 predictive techniques were evaluated in training dataset using LOOCV approach. Employing marker genes of TEX-associated TAMs, we determined that RF algorithm was the most effective for developing the model, which was capable of accurately predicting immunotherapy response across different cancers. This model integrated the feature selection strengths and robust predictive capabilities of RF through an ensemble ML approach. The model’s effectiveness was confirmed through various evaluation metrics, covering the confusion matrix, ROC curves, AUC values, calibration curves, and DCA curves. These metrics underscore its advantage over other clinical variables and highlight its significant potential for clinical decision-making. When compared to previous signatures for predicting immunotherapy efficacy, the model exhibited superior AUC values not only in the overall cohort but also in individual cancer-specific subgroups, indicating its broad applicability across different cancer types. Additionally, most of the genes in the model are distinct from the fourteen immune gene signatures previously mentioned. Risky gene of our model, SEMA6D (Semaphorin 6D), is a class VI trans-membrane semaphorin encoded on human chromosome 15q22.31. Initially identified as an axon-guidance cue, it now serves a broader role in tissue morphogenesis, angiogenesis and immune regulation. Researchers found that SEMA6D is predominantly expressed by non-hematopoietic cells, especially tumor cells, and shows a strong negative correlation with CD8A, PDCD1, IFNG and GZMB. Blocking its forward signaling therefore represents a promising strategy to enhance ICI efficacy (86). Meanwhile, protective gene of our model, CXCL9 regulates immune cell migration, differentiation, and activation, leading to tumor suppression (87). In sum, our model uniquely integrates SEMA6D-mediated immune evasion and CXCL9-driven anti-tumor immunity, offering a bi-directional molecular framework to refine risk stratification and boost ICI efficacy.

To gain deeper insights into the role of TAM-associated subset, we examined the STMN2+ TAM score specifically in NB using RNA-seq analysis. Our results revealed an important association across STMN2+ TAM scores and both immune dysfunction scores and T cell exhaustion scores. In parallel, scRNA-seq analysis highlighted a particularly notable difference in STMN2.SIG risk scores between responders and non-responders. The findings indicate that STMN2+ TAMs might play a substantial role in immunotherapy resistance. Numerous studies have demonstrated that different TAM subsets can influence T cell function and impact TIME to combat tumors. Additionally, our research underscores the connection between STMN2+ TAMs and CD8+ Tex cells, further supported by spatial trajectory analyses and spatially cellular interplay. Prior studies have shown that the spatial distribution of immune cells within TIME can affect biological processes in cancer patients. For instance, it has been observed that the distance among cancer cells and CD8+ PD-1+ T cells tends to expand following immunotherapy (88). Moreover, the spatial relationship between CD8+ T cells and cancer cells is linked to survival outcome, with greater separation being associated with a better prognosis (89). As for our research, we found that the distance between STMN2+ TAMs and CD8+ Tex cells was closer in non-responders compared to responders, suggesting a higher likelihood of cellular interplay across STMN2+ TAMs and CD8+ Tex cells in non-responders. Such interactions potentially led to immune evasion and T cell exhaustion through chemokine and cytokine networks, immune checkpoint molecules, metabolic reprogramming, and chronic antigen stimulation (90). Conversely, their association with functional T cells in responders indicates that the role of STMN2^+^ TAMs may be context-dependent. This stark contrast in cellular neighborhoods underscores the functional plasticity of STMN2^+^ TAMs and highlights their spatial positioning as a key determinant of immunotherapy efficacy. Targeting these specific cellular interactions could therefore represent a promising strategy to reverse T cell exhaustion and improve clinical outcomes.

Given that our study indicated the critical function of TEX-related TAMs in immunotherapy effectiveness, we delved deeper into their marker gene BCAP31 and explored how it might affect anti-tumor immunity. Our experimental results further indicated that TEX-related BCAP31+ TAMs, which are closely associated with CD8+ Tex cells, might play a role in anti-tumor immunity of NB. Consequently, we conducted *in vitro* co-culture experiments involving primary cancer cells, TAMs, and CD8+ T cells, demonstrating that silencing BCAP31 in TAMs results in increased cytokine production and activation of CD8+ T cells, potentially leading to immunotherapy responsiveness. Additionally, we aimed to uncover the mechanisms by which BCAP31 influences the malignant behaviors of tumor cells. Through GSEA analysis and WB validation, we demonstrated that downregulation of BCAP31 in TAMs is associated with inactivation of JAK2-STAT3 signaling pathway in tumor cells. We hypothesize that BCAP31 in TAMs may modulate JAK2-STAT3 signal in tumor cells, affecting the tumor progression. This inactivation of JAK2-STAT3 signal in tumor cells could result in decreased proliferating gene activation, ultimately contributing to tumor cell killing in NB.

The TIME, comprising tumor cells, stromal cells, blood vessels, nerve fibers, extracellular matrix, and related non-cellular components, exerts significant and diverse effects on the development, progression, and treatment response of cancer (91–94). Tumor infiltrating lymphocytes (TILs), a vital part of this microenvironment, show differing levels and conditions across various types of cancer (95). Emerging evidence suggests that TEX originates from a chronic state of dysfunction in TILs, encompassing a spectrum of phenotypic and transitional functional situations. Thus, understanding the dysregulation and exhaustion of T cells within TIME is essential for surmounting the tumor’s immune evasion mechanisms and boosting the effectiveness of immunotherapy in clinical settings. However, a comprehensive examination of the heterogeneity of TEX within the TIME of NB has been lacking. In our study, we meticulously investigated the varied composition of TEX within the NB TIME by leveraging TEX-related pathway signatures and genes sourced from databases. The single gene-defined marker for TEX, due to its lack of precision and quantitative limitations, proved insufficient in precisely depicting the progressive and hierarchical nature of TEX. In contrast, based on integrative scRNA-seq and stRNA-seq analyses, our findings notably captured the heterogeneity of TEX in a progressive and nuanced manner (13, 96).

The International Neuroblastoma Staging System (INSS) is currently utilized to guide treatment strategies and assess the prognosis of NB. Additionally, NB patients are frequently categorized into high-, intermediate-, or low-risk groups based on the Children’s Oncology Group (COG) risk classification. Nevertheless, neither the INSS nor the COG classification fully captures the highly variable clinic outcomes of NB. In our research, we defined marker genes of TEX-associated TAMs and utilized ML to develop NB-specific STMN2.SIG for prognostic prediction in NB patients. When this signature was applied to the train and test datasets, it effectively segregated NB cases into two distinct cohorts with markedly divergent survival outcomes, underscoring the model’s efficacy and dependability. The signature surpasses both INSS stage and COG risk in prognosticating survival. Furthermore, the signature has proven to be an independent and resilient instrument for evaluating OS. Importantly, when this risk score was applied to cases with MYCN amplification or those classified as high-risk by COG, as well as patients in immunotherapy cohorts, it successfully stratified these patients into high and low-risk categories. This classification will assist in evaluating the survival prospects of these high-risk cases before they undergo potentially harmful treatments, thereby helping to avert long-term adverse effects.

Our STMN2.SIG could be smoothly employed with qPCR detection methods, making it a viable option for widespread clinical use. However, it is essential to acknowledge certain limitations inherent in our study. Firstly, our research was conducted retrospectively, utilizing sequencing data and associated clinical information sourced from public repositories. The absence of detailed therapeutic protocols, metastatic sites, and recurrence data might potentially affect our results. Secondly, the role of BCAP31 in NB remains to be fully elucidated, as the direct intercellular mediator was not identified among tumor cells, macrophages and CD8+ T cells. Consequently, further research involving additional tumor samples and more in-depth experimental studies for intercellular cross-talk is needed to explore its biological functions within NB. Another limitation lies in the scope of our spatial cellular interactions, which was performed on a dataset from HCC. Future validation using stRNA-seq datasets from other malignancies is warranted to assess the generalizability of this finding. Lastly, our current model development approach, which relies solely on transcriptome sequencing, would benefit greatly from the integration of multi-omics and multi-modal data. This comprehensive integration allows for a more detailed understanding of the molecular mechanisms and physiological processes, thereby enhancing the reliability and accuracy of predictive models. The incorporation of multi-omics and multi-modal data introduces a multitude of variables into the analysis, which is crucial for developing more sophisticated artificial intelligence models. Therefore, deep learning, a subset of ML, has the distinct capability to autonomously detect key features for classification. This is not easily achievable with traditional ML methods, which necessitate the manual identification and input of these features. As a result, the implementation of cutting-edge deep learning algorithms, combined with the valuable insights gained from integrating multi-omics and multi-modal data, represents a potent approach to advancing personalized medicine for NB patients.

## Conclusion

In our study, we firstly pioneer the usage of large-scale pan-cancer scRNA-seq and RNA-seq immunotherapy datasets to develop and validate the pan-cancer STMN2.SIG for predicting immunotherapy response through ML approaches. Moreover, we investigate the underlying mechanisms in NB that enable the STMN2.SIG to delineate immune landscapes, specifically, the interaction between STMN2+ TAMs and CD8+ Tex cells. The downregulation of BCAP31 on TAMs could remarkably inhibit the malignant behaviors of NB cells and promote the effector function of CD8+ T cells in TIME. BCAP31 on TAMs is associated with the modulation of JAK2-STAT3 pathway in tumor cells, indicating the BCAP31-targeted therapy in TAMs may present promising therapeutic strategies.

## Data Availability

The datasets presented in this study can be found in online repositories. The names of the repository/repositories and accession number(s) can be found below: https://github.com/douzoulle/pan_ICI_NB.
