# Supplementary material for: Novel pan-cancer T cell exhaustion signature forecasts immunotherapy response and unveils BCAP31 in macrophages as a therapeutic target in neuroblastoma
Source: Front Immunol. 2025 Dec 22;16:1709225. doi: 10.3389/fimmu.2025.1709225 (PMC12721133; doi:10.3389/fimmu.2025.1709225)
Supplement: Supplementary file 1 [file DataSheet1.docx]

**Novel Pan-cancer T Cell Exhaustion Signature Forecasts Immunotherapy Response and Unveils BCAP31 in Macrophages as a Therapeutic Target in Neuroblastoma**

**Shan Li# 1,4, Jianjun Zhu# 2, Xiang Huang# 2, Fengming Ji 1, Jinrong Li 1, Zhigang Yao 1, Haoyu Tang 1, Ling Liu* 5, Bing Yan* 1,3, Chenghao Zhanghuang#* 1,3,4**

**Supplemental Figures 1-7**

**Supplemental Table 1-3**

Supplementary Figure 1

A) Different cell proportions of immunotherapy outcomes in scRNA-seq cohorts. B) Comparison of immune evasion and immune checkpoint genes among major cell types. C) Pseudotime DEGs identified by pseudotime analysis. D) DEGs of TAMs in responders and non-responders. E) Distinction expression profiles of TAMs between responders and non-responders.


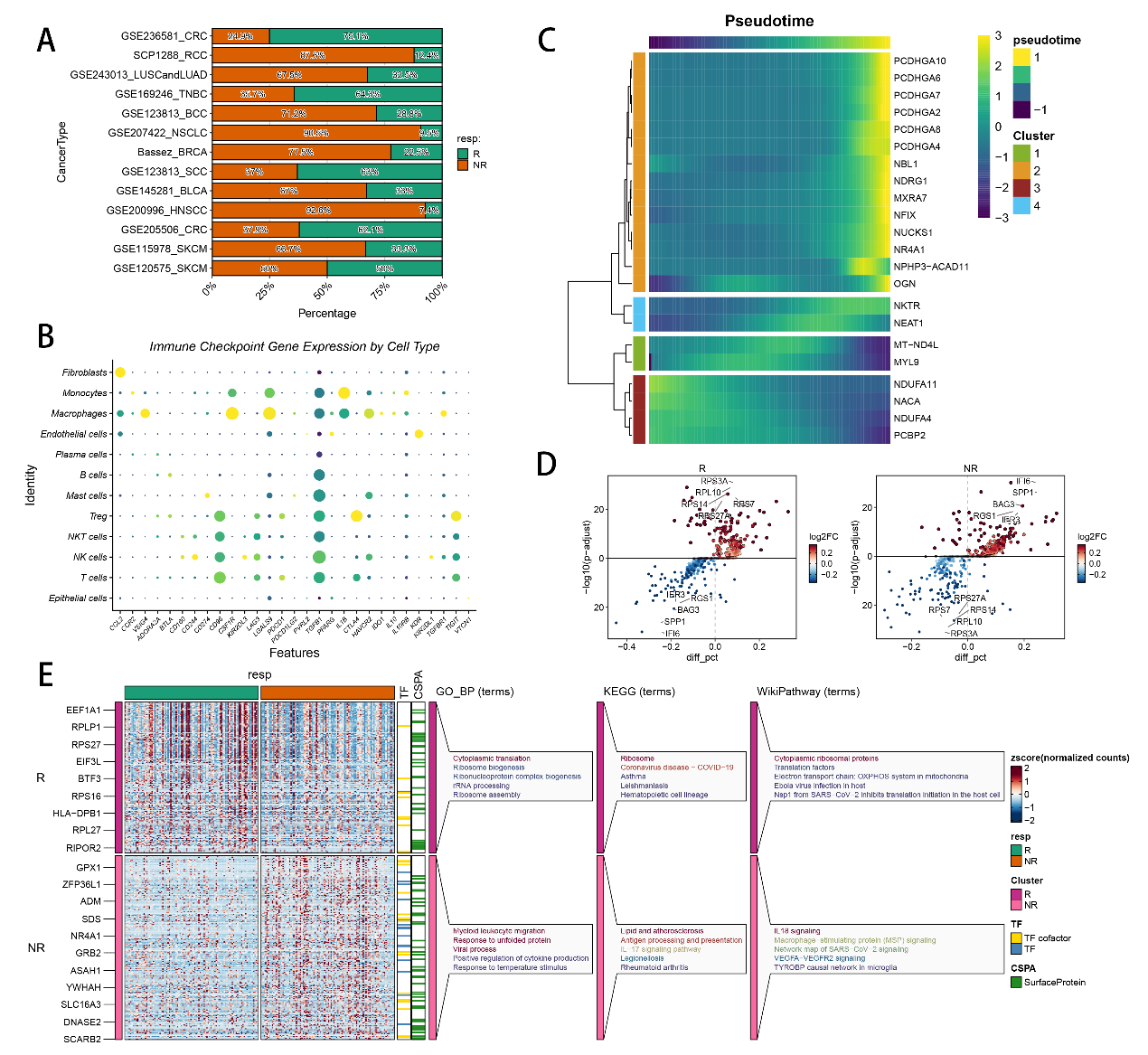


Supplementary Figure 2

A) Visualizing marker genes of each TAM subtype by dotplot. B) Visualizing marker genes of each TAM subtype by heatmap, as well as enrichment analysis results of each TAM subtype by GO and KEGG. C) Expression of immune evasion and immune checkpoint genes in TAM subpopulations. D) Utilizing scRNA-seq scoring algorithm (ssGSEA, GSVA, AddModuleScore, and PercentageFeatureSet) to validate the top TEX enrichment scores in STMN2+ TAMs. E) Comparison of transcription factor activities among TAM subclusters.


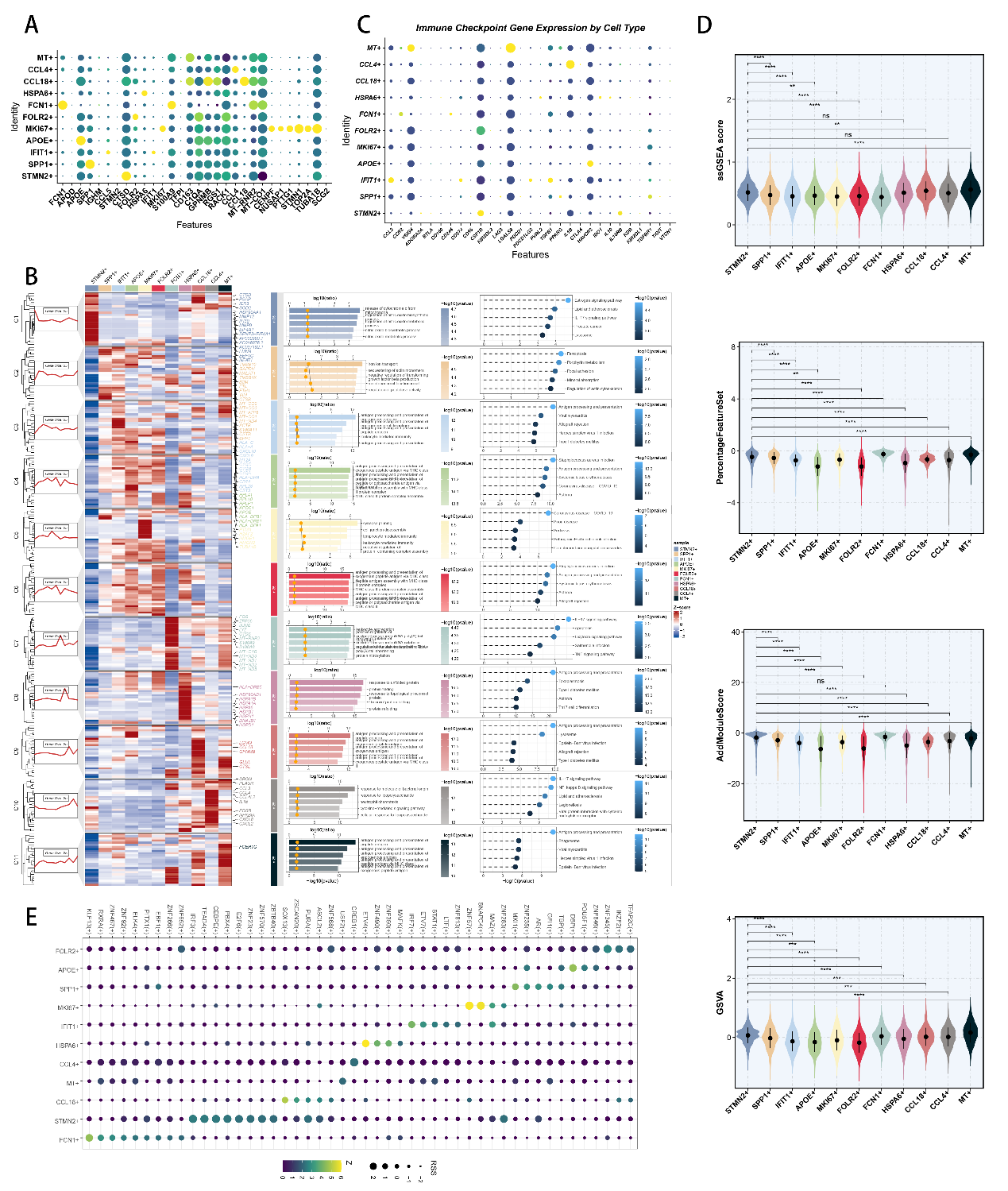


Supplementary Figure 3

A) Distinct overall signaling patterns in responders and non-responders among T cell subgroups and TAM subgroups. B) Ligands and receptors for signal communication among STMN2+ TAM and different T cell subpopulations. C) DEGs of of CD8+ Tex cells between responders and non-responders. D) Distinct expression profiles of CD8+ Tex cells between responders and non-responders. E) Exhausted scores of CD8+ Tex cells in UMAP by AUCell and ssGSEA algorithm. F) Effector scores of Tem/Teffe cells in UMAP by AUCell and ssGSEA algorithm.


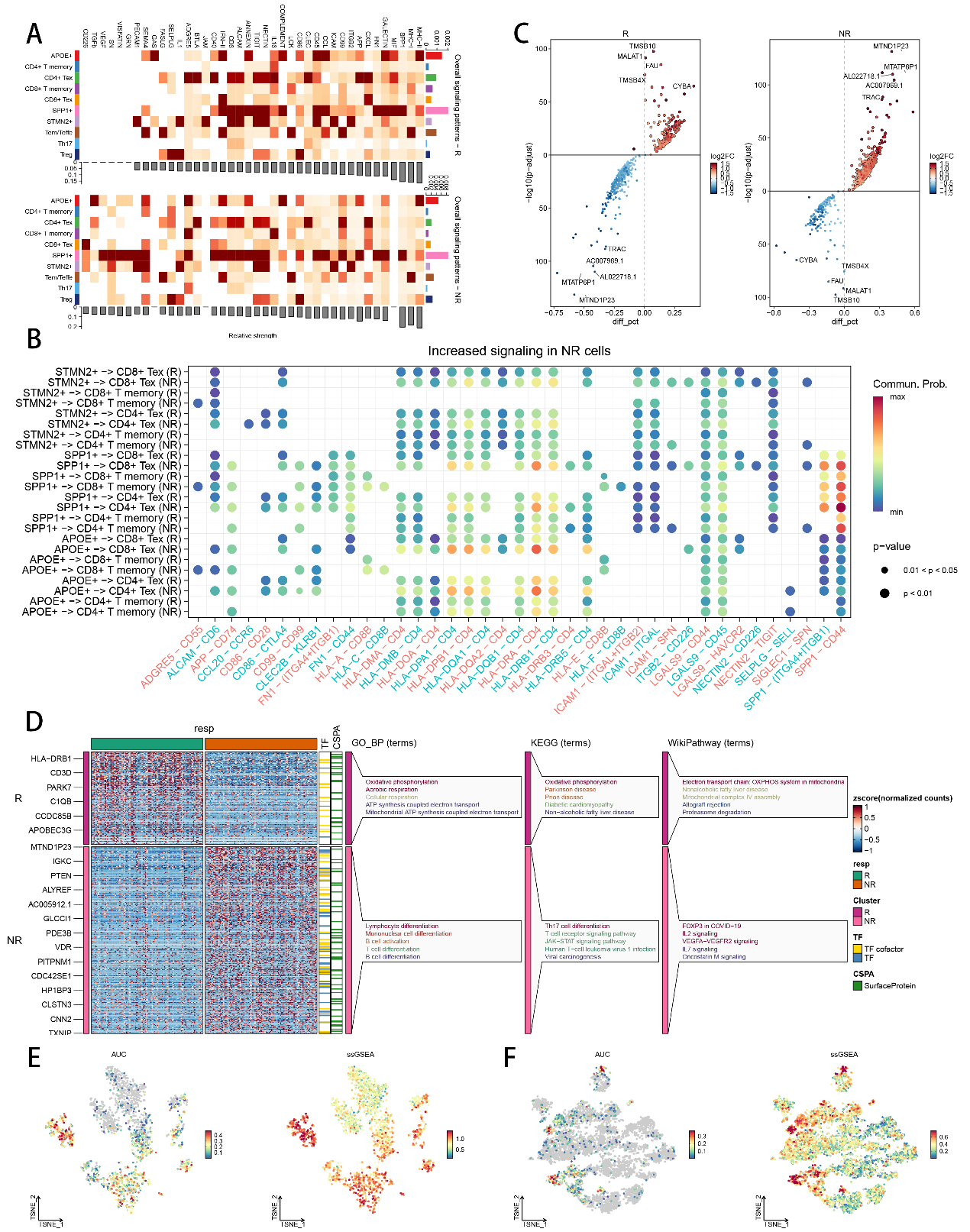


Supplementary Figure 4

A) Violin plots show the quality control feature of percentage of mitochondrial genes in each patient. B) Unbiased clustering of stRNA-seqx spots and cell types of each cluster in each patient. C) High-density regions of every cell subpopulation in each tumor section.


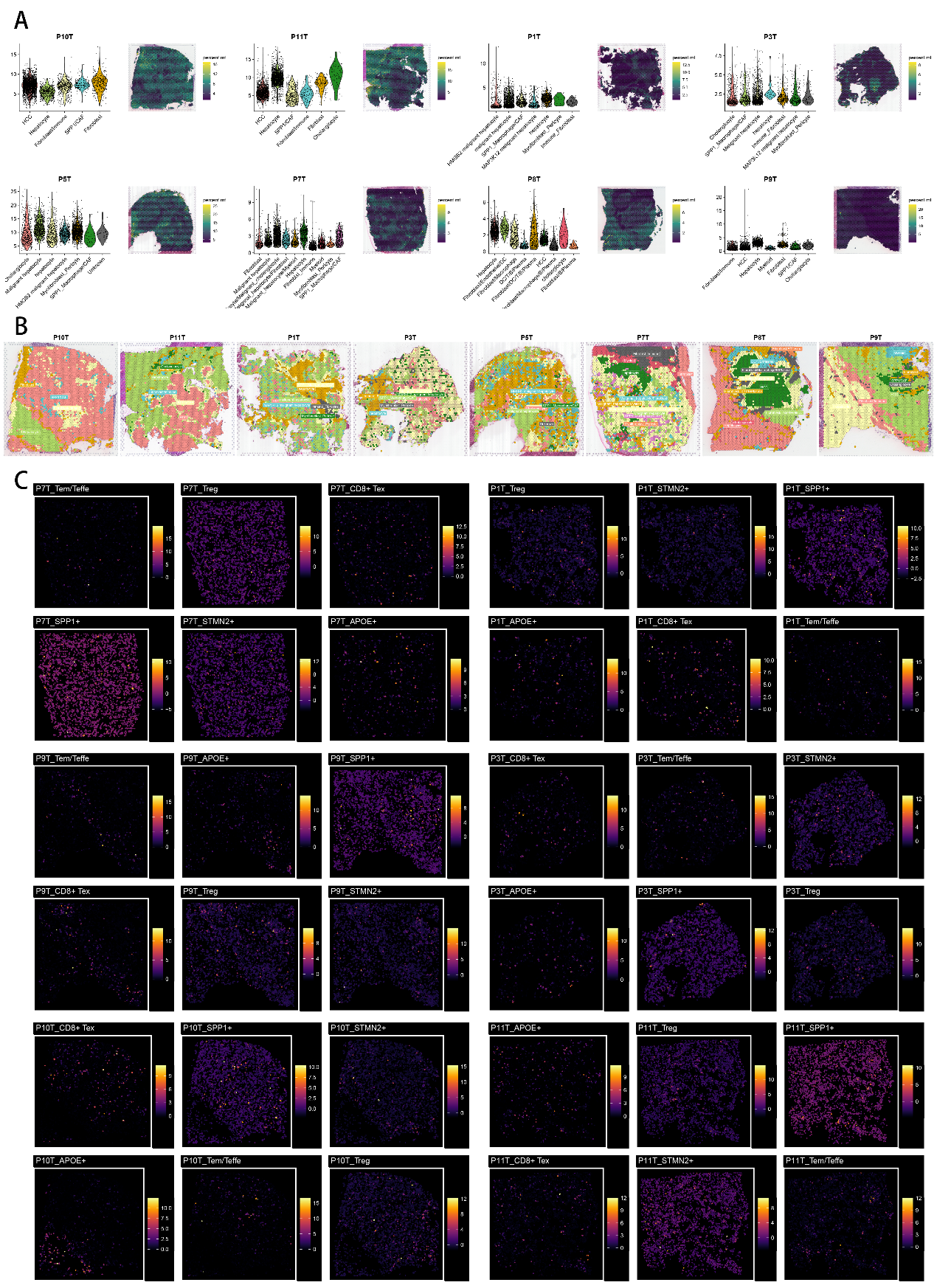


Supplementary Figure 5

A) The interplay distances of LGALS1-PTPRC between STMN2+ TAMs and CD8+ Tex in non-responders and responders. B) The interplay distances of LGALS1-PTPRC between STMN2+ TAMs and Tem in non-responders and responders. C) Integrated ranking of ligand-receptor interactions based on interplay distances between STMN2+ TAMs and CD8+ Tex using RRA algorithm in responders and non-responders. The smaller the RRA score of a certain ligand-receptor interaction, the closer it is between STMN2+ TAMs and CD8+ Tex.


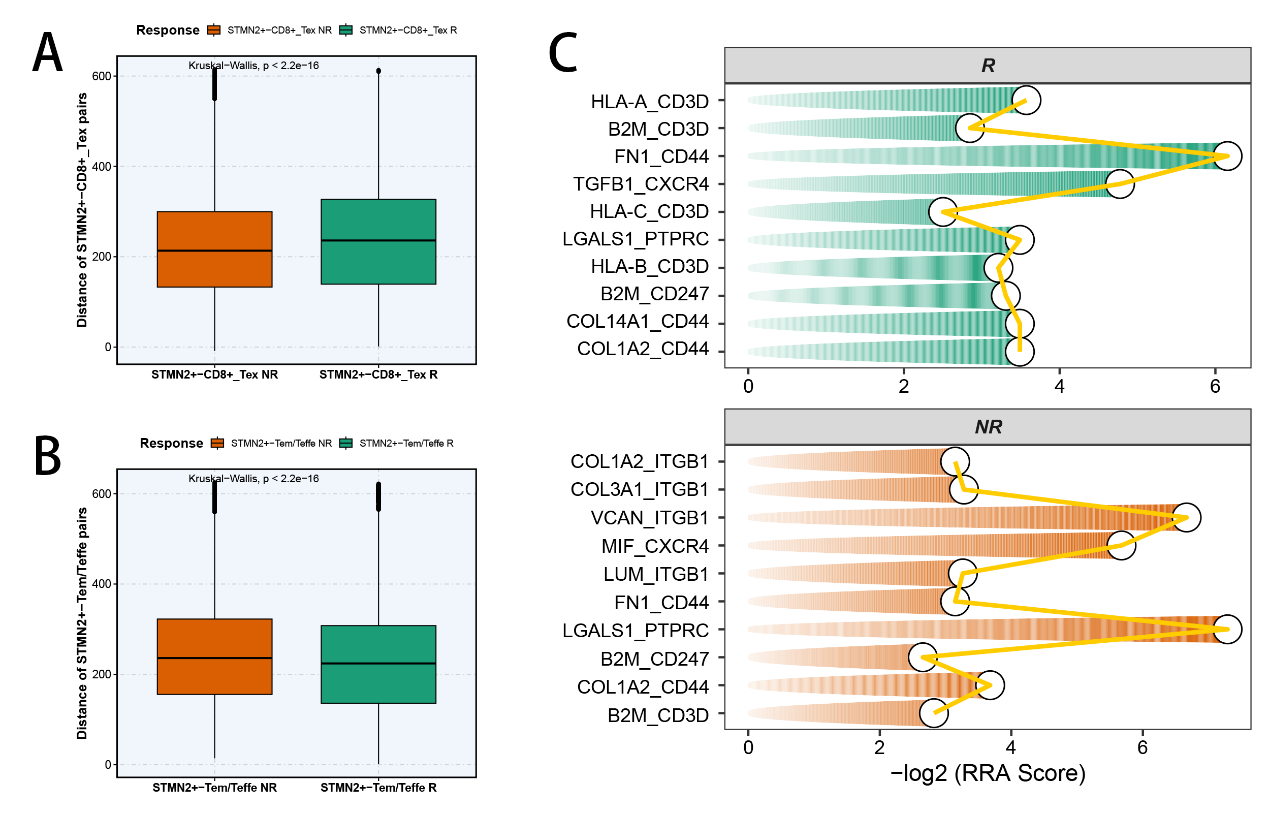


Supplementary Figure 6

A) UMAP plot of canonical markers of TAM subtypes in NB scRNA-seq cohort. B) Violin plot of canonical markers of TAM subtypes in NB scRNA-seq cohort. C) Subclusters of TAMs at resolution 0.1 in NB scRNA-seq cohort. D) Cell cycle scoring of TAMs in NB scRNA-seq cohort. E) Visualizing marker genes of each neuroendocrine cell subtype by heatmap, as well as enrichment analysis results of each neuroendocrine cell subtype by GO and KEGG.


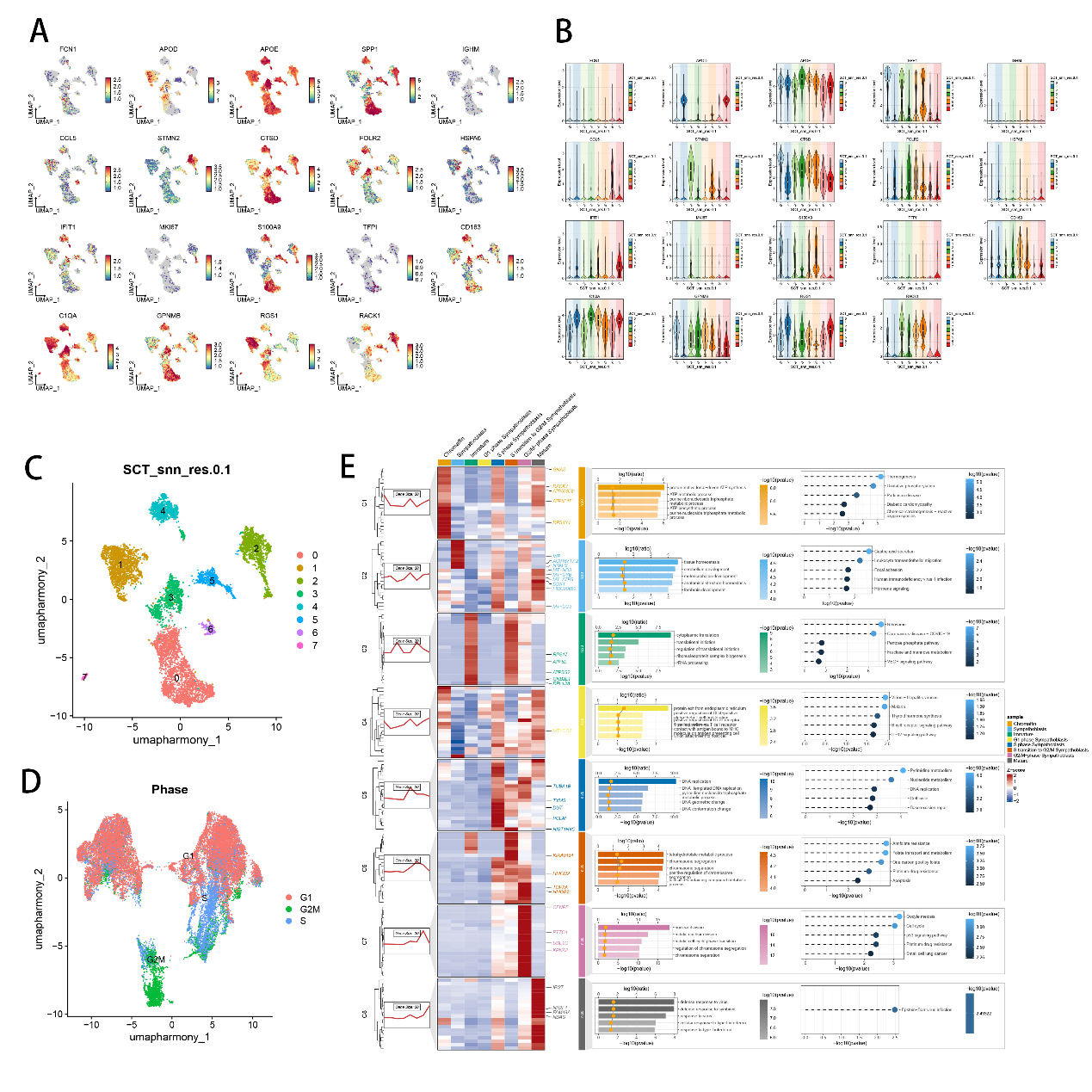


Supplementary Figure 7

A) PCA plots visualizing data integration before and after batch correction. B) Comparison of the AUCs among the STMN2.SIG model and other published signatures in RCC combined dataset. C) Confusion matrix of the STMN2.SIG Model in the training, internal validation, and external validation cohorts. D) Comparing STMN2.SIG risk scores with TMB and PDL-1 based on ROC Curves in IMmotion151, Mariathasan, Braun, and Riaz cohorts. E) Multivariate logistic regression analysis in IMmotion151, Mariathasan, Braun, and Riaz cohorts. F) AUC of the STMN2.SIG Model, several clinical variables and the nomogram model in the Braun cohort. G) Calibration curves in the training, internal validation, and external validation cohorts. H) DCA curves of the STMN2.SIG Model, several clinical variables and the nomogram model in the Braun cohort. I) The nomogram illustration of the STMN2.SIG model in the Braun cohort.


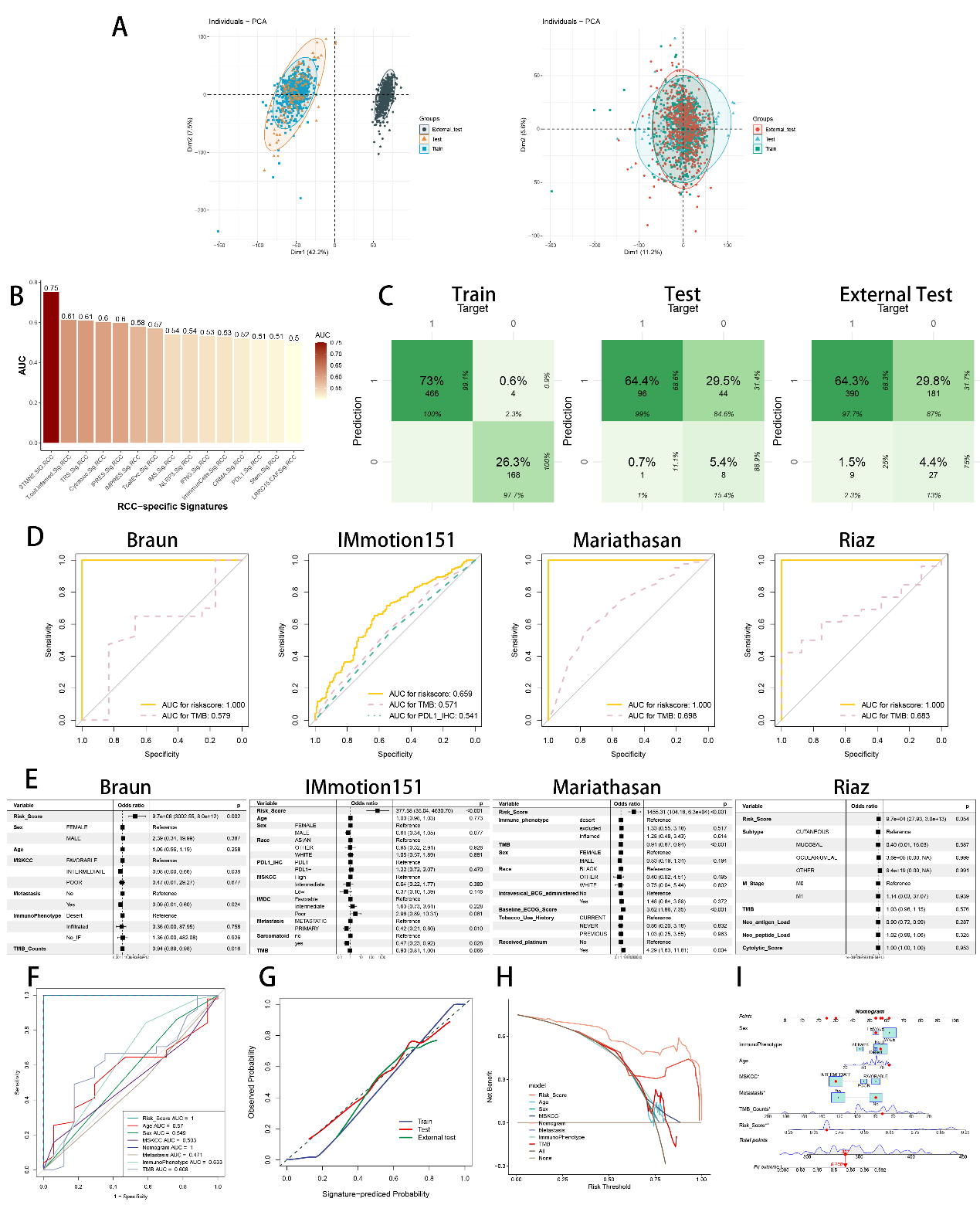


**Supplemental Table 1: The siRNA targeted sequence used in this study.**

| **Oligonucleotides** | **Sequence(5’-3’)** |
| --- | --- |
| siBCAP31-1 | CUCAGAGGAAUCUCUAUAUTT |
| siBCAP31-2 | GAGGGCCUUACCAAAGAAUTT |
| siBCAP31-3 | CCUCCAAUGAAGCCUUUAATT |

**Supplemental Table 2: The primers used in this study.**

| **Primers** | **Sequence (5’-3’)** |
| --- | --- |
| BCAP31 forward | CCTCTATGCGGAGGTCTTTGT |
| BCAP31 reverse | CCGTCACATCATCATACTTCCGA |
| GAPDH forward | TGTCACACGCTTTTGGGGTTT |
| GAPDH reverse | CCTGGAAGATGGTGATGGGATT |

**Supplemental Table 3: The Flow Cytometry antibodies used in this study.**

| **Brand** | **Item Number** | **Product Name** |
| --- | --- | --- |
| BioLegend | 423102 | Zombie Aqua™ Fixable Viability Kit |
| BioLegend | 304062 | APC/Fire™ 750 anti-human CD45 |
| BioLegend | 317306 | FITC anti-human CD3 |
| BioLegend | 344710 | PerCP/Cyanine5.5 anti-human CD8 |
| BioLegend | 345008 | Brilliant Violet 421™ anti-human CD366 (Tim-3) |
| BioLegend | 329908 | APC anti-human CD279 (PD-1) |
| BioLegend | 369208 | PE/Cyanine7 anti-human CD223 (LAG-3) |
| BioLegend | 349906 | PE anti-human CD152 (CTLA-4) |
| BioLegend | 502532 | Brilliant Violet 421™ anti-human IFN-γ |
| BioLegend | 372204 | APC anti-human/mouse Granzyme B Recombinant |
| BioLegend | 502930 | PE/Cyanine7 anti-human TNF-α |
| BioLegend | 308103 | FITC anti-human Perforin |
| BioLegend | 308106 | PE anti-human Perforin |
